# The Therapeutic Potential of Exosomes vs. Matrix-Bound Nanovesicles from Human Umbilical Cord Mesenchymal Stromal Cells in Osteoarthritis Treatment

**DOI:** 10.3390/ijms252111564

**Published:** 2024-10-28

**Authors:** Timofey O. Klyucherev, Maria A. Peshkova, Daria P. Revokatova, Natalia B. Serejnikova, Nafisa M. Fayzullina, Alexey L. Fayzullin, Boris P. Ershov, Yana I. Khristidis, Irina I. Vlasova, Nastasia V. Kosheleva, Andrey A. Svistunov, Peter S. Timashev

**Affiliations:** 1 Institute for Regenerative Medicine, I. M. Sechenov First Moscow State Medical University, 119991 Moscow, Russia; 2 Laboratory of Clinical Smart Nanotechnologies, Institute for Regenerative Medicine, Sechenov University, 119991 Moscow, Russia; 3 Laboratory of Digital Microscopic Analysis, Institute for Regenerative Medicine, Sechenov University, 119991 Moscow, Russia; 4 Sechenov First Moscow State Medical University, 119991 Moscow, Russia; 5 World-Class Research Center “Digital Biodesign and Personalized Healthcare”, I. M. Sechenov First Moscow State Medical University, 119991 Moscow, Russia

**Keywords:** osteoarthritis, mesenchymal stromal cells, exosomes, matrix-bound nanovesicles, monocyte-derived macrophages

## Abstract

Osteoarthritis (OA) is a degenerative joint disease with limited therapeutic options, where inflammation plays a critical role in disease progression. Extracellular vesicles (EV) derived from mesenchymal stromal cells (MSC) have shown potential as a therapeutic approach for OA by modulating inflammation and alleviating degenerative processes in the joint. This study evaluated the therapeutic effects for the treatment of OA of two types of EV—exosomes and matrix-bound nanovesicles (MBV)—both derived from the human umbilical cord MSC (UC-MSC) via differential ultracentrifugation. Different phenotypes of human monocyte-derived macrophages (MDM) were used to study the anti-inflammatory properties of EV in vitro, and the medial meniscectomy-induced rat model of knee osteoarthritis (MMx) was used in vivo. The study found that both EV reduced pro-inflammatory cytokines IL-6 and TNF-α in MDM. However, exosomes showed superior results, preserving the extracellular matrix (ECM) of hyaline cartilage, and reducing synovitis more effectively than MBVs. Additionally, exosomes downregulated inflammatory markers (TNF-α, iNOS) and increased Arg-1 expression in macrophages and synovial fibroblasts, indicating a stronger anti-inflammatory effect. These results suggest UC-MSC exosomes as a promising therapeutic option for OA, with the potential for modulating inflammation and promoting joint tissue regeneration.

## 1. Introduction

OA is a complex joint disease affecting various joint tissues, and in the progression of OA, impaired mechanical properties of the meniscus, subchondral bone, as well as the development of fibrosis in the infrapatellar fat pad (IFP) and the synovial membrane play an important role [1,2,3,4,5]. The incidence of OA is rapidly increasing due to population aging, and it has currently reached a prevalence of 7% worldwide, being a common cause of disability and decreased quality of life [2]. OA was traditionally considered a non-inflammatory arthritis resulting from mechanical cartilage damage [6]. However, several studies have shown that one of the important factors in OA pathogenesis is inflammation, which develops in response to chondrocyte death and degradation of ECM components of cartilage tissue [7]. Inflammation in OA is significantly influenced by the tissues of the synovial membrane and infrapatellar fat pad (IFP), which secrete pro-inflammatory mediators, such as adipokines and cytokines, creating an inflammatory microenvironment in joint tissues during OA, significantly affecting the enhancement of degenerative processes in cartilage tissue [4,8,9]. Damage-associated molecular patterns (DAMP) released during cartilage damage trigger inflammatory activation of synovial and IFP fibroblasts and macrophages, leading to the secretion of inflammatory factors such as tumor necrosis factor-alpha (TNF-α), interleukins (IL) IL-1, IL-6, and matrix metalloproteinases (MMPs) [10,11,12]. These inflammatory cytokines and proteases exacerbate cartilage and subchondral bone damage, leading to functional limitations of the joint [13].

One of the key challenges in OA therapy is the lack of disease-modifying drugs capable of slowing the disease’s progression [7,14]. Currently, pharmacotherapy primarily aims to alleviate OA symptoms, with anti-inflammatory drugs such as nonsteroidal anti-inflammatory drugs (NSAID) and glucocorticoids used for this purpose [15,16]. These medications relieve the main symptoms of pain and swelling and improve joint mobility; however, they do not significantly slow the degenerative changes in cartilage tissue and have notable side effects [17]. In cases of significant OA progression, surgical interventions such as knee or hip joint replacement, subchondral bone microfracture, osteochondral allograft/autograft transplantation, and autologous chondrocyte transplantation are used [18]. Surgical interventions demonstrate significant effectiveness, particularly joint replacement of the OA-affected joint, but these interventions are costly, require long rehabilitation periods for OA patients, and do not always restore the anatomically normal structure of hyaline cartilage [19,20].

The disadvantages of existing approaches include their low specificity and the lack of targeting of signaling pathways associated with OA progression. In recent decades, cell therapy for OA, based on the transplantation of autologous chondrocytes, has gained widespread popularity [21]. Despite the therapeutic effectiveness and good safety profile, one of the serious limitations of such therapy is the limited capacity of chondrocytes for differentiation and the loss of phenotype during in vitro expansion, which prevents this approach from finding broad application in OA therapy [21,22]. An alternative direction in cell therapy for OA involves the use of mesenchymal stromal cells (MSC) [20]. MSC possess high regenerative potential due to their ability to differentiate and proliferate, as well as their immunomodulatory activity [23]. Thanks to their ability to regulate the inflammatory process, MSC hold great promise in the treatment of degenerative diseases, including OA [24,25]. Bone marrow-derived MSC can mitigate degenerative processes in cartilage during OA by reducing inflammation in the synovial membrane and attenuating catabolic processes in chondrocytes [26,27]. MSC therapy is relatively safe, but questions remain regarding their immunogenicity, as well as the risks of thrombosis and tumor transformation when administered in vivo [28,29]. There is now an understanding that MSC possess regenerative potential through paracrine activity, thanks to various secreted factors, including extracellular vesicles (EV) [30,31,32].

EV are one of the main therapeutic components of the MSC secretome. Among the EV secreted into the surrounding environment, several subclasses can be distinguished, such as exosomes, microvesicles, and apoptotic bodies. EV differ in size, markers, and the method of biogenesis [33,34]. Due to exosomes diverse cargo, such as regulatory RNA molecules, proteins, and lipids, exosomes can modify the phenotypes of immune cells, including T and B lymphocytes, macrophages, and dendritic cells, as well as modulate gene expression in chondrocytes and osteoblasts, enhancing their survival and proliferation [35,36,37]. In addition to EV from the secretome, researchers have identified EV associated with extracellular matrix components, namely matrix-bound nanovesicles (MBV) [38]. It is believed that MBV are a subtype of microvesicles embedded within the ECM components of soft tissues, with an average size of 20–200 nm, and they practically do not express endosomal markers typical of exosomes, such as CD9, CD63, and CD81 [39,40]. In most studies, MBV have been identified and isolated from the ECM of the bladder and small intestine submucosa. In some studies, MBV were isolated from monolayer cultures of 3T3 fibroblasts and human MSC [38,41,42]. Several studies have demonstrated the remarkable therapeutic potential of MBV, primarily due to their immunomodulatory activity through the shift of macrophages from a pro-inflammatory M1 phenotype to an anti-inflammatory M2 phenotype [43,44]. This property of MBV makes these vesicles a potential therapy for OA, as increased M1 macrophage activity plays a key role in the development of synovial inflammation in OA [45]. Alongside exosomes, MBV derived from MSC could potentially have a high therapeutic potential in managing the inflammatory process in OA [35].

One of the common cellular models of EV therapy for OA involves chondrocytes treated with IL-1β, where exosomes from bone marrow MSC and other sources reduced apoptosis and catabolic processes in chondrocytes and enhanced chondrocyte migration, survival, and proliferation [46,47]. In a study of monoiodoacetate (MIA)-induced OA therapy using exosomes from amniotic fluid stem cells, exosomes reduced the expression of markers associated with M1 polarization (CD86 and IL-1R1) in M1 THP-1 macrophages and increased CD163 and arginase 1 markers associated with the M2 macrophage phenotype [48]. These exosomes also contributed to the reduction in destructive processes in the cartilage tissue of the knee joints in rats with MIA-induced OA by decreasing the expression of inflammation-related proteins Inducible nitric oxide synthase (iNOS) and increasing Sox9 expression, which is associated with anabolic processes in hyaline cartilage chondrocytes [48]. UC-MSC exosomes demonstrate a pronounced anti-inflammatory effect by reducing the secretion of the inflammatory cytokine IL-1β and NLRP3 [49]. Additionally, this study showed the therapeutic efficacy of UC-MSC-derived exosomes in an OA animal model induced by medial meniscus destruction, promoting the increase in ECM repair-related proteins collagen type II, alpha 1, Aggrecan in hyaline cartilage and reducing proteases (ADAMTS5 and MMP13) associated with ECM degradation during OA progression [49].

The immunomodulatory properties of MSC-EV are influenced by a number of factors, including cultivation conditions. For example, under hypoxia, preconditioning with pro-inflammatory cytokines enhanced the regenerative potential of MSC-EV in the treatment of various inflammatory diseases, including OA [50,51]. The protocol of nanoparticle isolation has a strong effect on the therapeutic properties of exosomes obtained from MSC [52]. The use of FBS for MSC cultivation can negatively affect the properties of EV due to contamination with FBS-derived components in the EV samples. However, serum-free media may influence the cargo of EV as well as their biological properties [53]. One of the challenges in studying the therapeutic effects of MSC-derived EV cultured in FBS-free media and isolated using differential ultracentrifugation is the unwanted contamination with stress-related proteins and media components. These contaminants may negatively impact the regenerative and anti-inflammatory properties of MSC-derived exosomes in cellular and animal models of OA [52,54].

The human umbilical cord MSC (UC-MSC) is one of the optimal sources of MSC-derived EV, as these cells have a strong ability to modulate inflammation, proliferation, and differentiation [36,55]. The use of UC-MSC-derived EV in the treatment of degenerative and inflammatory diseases demonstrates the high therapeutic potential of UC-MSC EV, making this source of EV a promising therapy for OA [49,56]. In this study, both types of EV, exosomes and matrix-bound nanovesicles derived from UC-MSC were used to evaluate the therapeutic potential of EV in OA therapy. The anti-inflammatory properties of EV were studied in vitro by the use of human MDM of various phenotypes. In the animal model of OA, the surgical medial meniscectomy-induced (MMx) in vivo knee osteoarthritis rat model was employed. On day 21 of OA induction, the animals received an intra-articular injection of MBV and UC-MSC exosomes, followed by histological evaluation of the efficacy of EV therapy on days 7 and 21.

## 2. Results

### 2.1. Immunophenotype Analysis of UC-MSC and Visualization of MBV and Exosomes from UC-MSC

**Figure 1:**
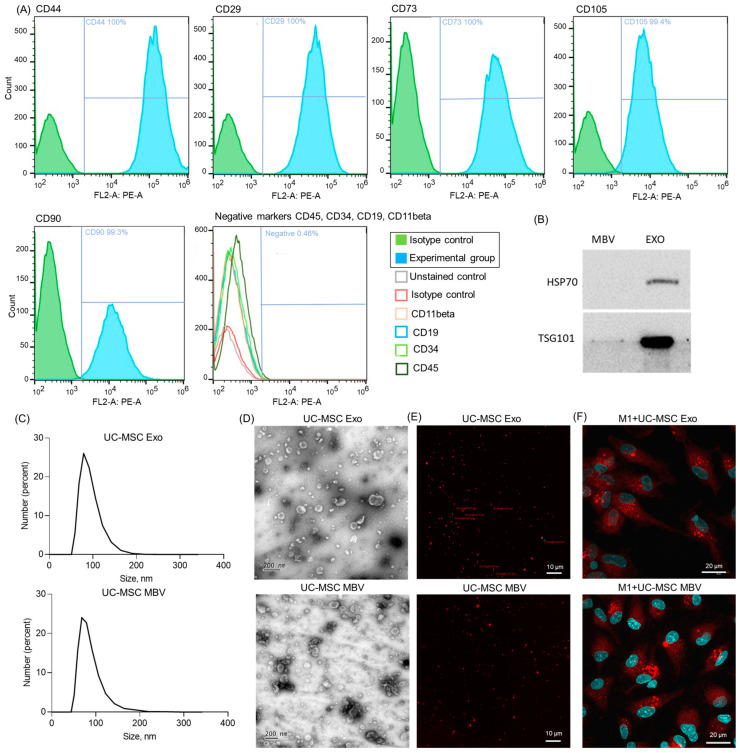
Characterization of UC-MSC exosomes and UC-MSC MBV. (**A**) Flow cytometry results show the presence of phenotypic markers for UC-MSC (CD105, CD73, CD90, CD29, CD44) and the absence of hematopoietic markers (CD19, CD11beta, CD45, CD34). (**B**) Western blot analysis of UC-MSC Exo for the presence of exosomal markers (HSP70+, TSG101+). (**C**) Particle size distribution obtained by nanoparticle dynamic light scattering. (**D**,**E**) Visualization of UC-MSC Exo and UC-MSC MBV via TEM and confocal microscopy with fluorescent staining of extracellular vesicles using PKH26. (**F**) Phagocytosis of UC-MSC Exo and UC-MSC MBV labeled with PKH26 by M1 (IFN-γ + LPS) macrophages.

Using flow cytometry, we demonstrated that UC-MSC express characteristic mesenchymal markers (CD105, CD73, CD90, CD29, CD44) with no significant expression of hematopoietic markers (CD19, CD11beta, CD45, CD34) (Figure 1A). Positive mesenchymal markers included CD105 (99.4%), CD73 (100%), CD90 (99.3%), CD29 (100%), and CD44 (100%) for UC-MSC, while the proportion of negative markers was no more than 0.46%. For the liquid-phase EV from the UC-MSC secretome, we will use the term exosomes (Exo), although it should be noted that liquid-phase EV may also contain microvesicles, as the size ranges of microvesicles and exosomes may overlap. Microvesicles could enter the sample using the protocol for isolating MBV from the UC-MSC secretome employed in this study. The isolated exosomes expressed the characteristic exosomal markers HSP70+ and TSG101+ (Figure 1B). In contrast, MBV lacked most exosomal markers; only a minimal amount of TSG101+ was detected in MBV samples, consistent with previous studies [38]. The results of the nanoparticle dynamic light scattering analysis showed that the obtained Exo and MBV had an average size of 40–150 nm, with MBV being approximately 78.8 nm ± 22.9 nm (mean± standard deviation) and exosomes slightly larger at 91.3 nm ± 21.7 nm (Figure 1C). Transmission electron microscopy (TEM) images revealed that both types of vesicles had a characteristic cup-shaped, rounded morphology, with particle sizes averaging 100–200 nm (Figure 1D). Both types of MBV were shown to contain lipid bilayers, as they were able to internalize the membrane layer dye PKH26, demonstrated via fluorescence microscopy (Figure 1E). Micrographs and graphs in the best resolution are presented in Supplementary Materials Figures S1–S5.

### 2.2. Phagocytosis of Extracellular Vesicles (Exosomes and MBV from UC-MSC) by Human Macrophages Leads to Modulation of Pro-Inflammatory Cytokine Secretion

Human macrophages derived from peripheral blood mononuclear cells (PBMC) were selected as an in vitro model for evaluating the immunomodulatory properties of UC-MSC Exo and MBV. The ability of UC-MSC EV to modulate the pro-inflammatory activity of monocyte-derived macrophages (MDM) was evaluated for four phenotypes: M0 cultured with GM-CSF (M0_GM), M0 cultured with M-CSF (M0_M), pro-inflammatory M1 macrophages (IFNγ + LPS), and reparative M2 macrophages (IL-4). All four macrophage types were previously characterized for polarization markers on the 8th day of culture [57]. M1 macrophages secreted high levels of pro-inflammatory cytokines and expressed CD86, while M2 macrophages were characterized by IL-10 secretion and CD206 expression. A flow cytometry analysis of MDM polarization markers CD86 and CD206 is presented in Supplementary Figure S6. All MDM phenotypes demonstrated typical macrophage phagocytic activity. The ability of macrophages to phagocytize EV was shown for exosomes and MBV labeled with the membrane dye PKH26, as demonstrated in M1 (Figure 1F).

**Figure 2:**
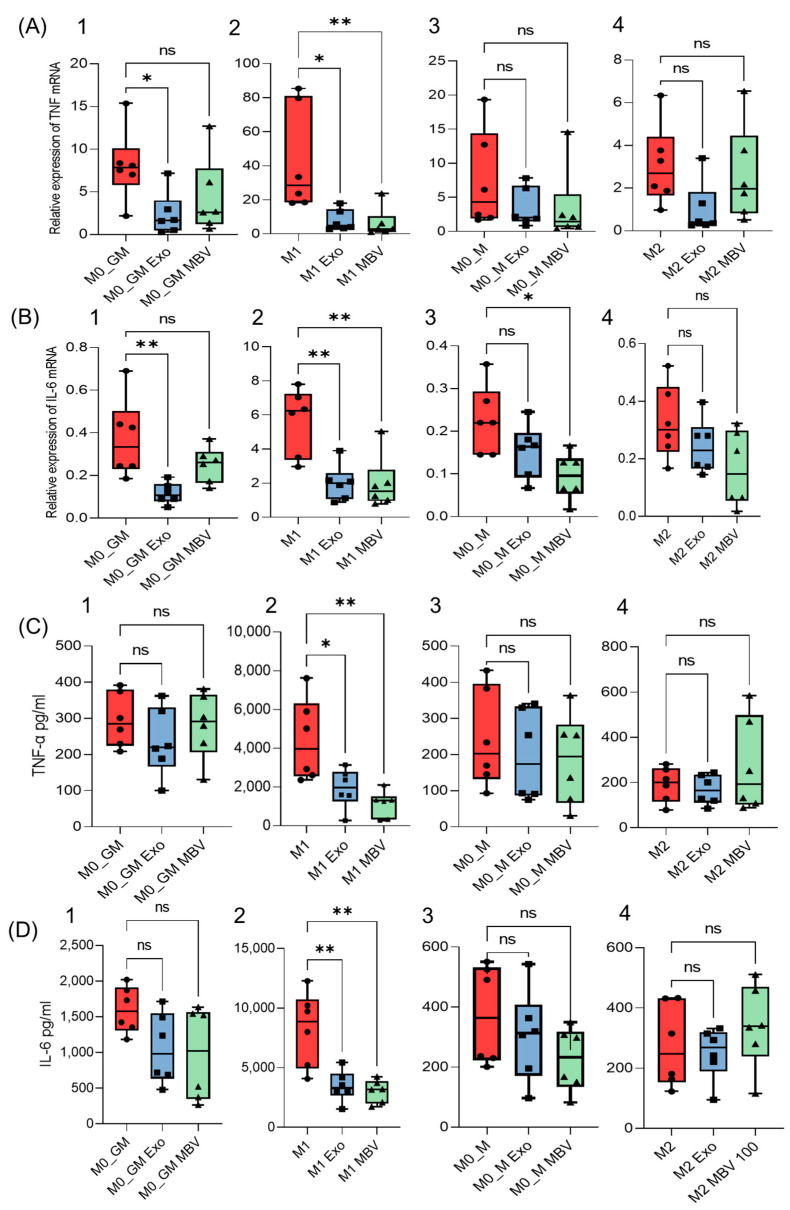
Modulation of pro-inflammatory cytokine levels in MDM by exosomes and MBV derived from UC-MSC. Results of qRT-PCR for (**A**)—TNFα, (**B**)—IL-6, and ELISA for (**C**)—TNFα, (**D**)—IL-6 for the following macrophage phenotypes: 1. M0_GM, 2. M1, 3. M0_M, 4. M2. * *p* < 0.05; ** *p* < 0.01; ns (*p* > 0.05). The figures (circle, square, triangle) on the graphs indicate the concentration of the studied protein or the level of mRNA expression in the experimental sample: ● (control group); ■ (experimental group + exosomes); ▲ (experimental group + MBV).

In our experiments, on day 6 of culture MDM, with growth factors M-CSF and GM-CSF after medium replacement, and the addition of polarization inducers for obtaining M1 (IFN-γ + LPS) and M2 (IL-4), UC-MSC MBV and UC-MSC exosomes were added to MDM at a concentration of 100 µg of vesicle protein per 1 mL of medium. After 48 h cultivation, the medium was collected to measure the production of pro-inflammatory cytokines TNFα and IL-6 (via ELISA), and cells were lysed to characterize cytokine mRNA expression (Figure 2). UC-MSC MBV and exosomes showed significant immunomodulatory activity against pro-inflammatory M1 macrophages, with both vesicle types significantly reducing both the expression and secretion of TNFα and IL-6 (Figure 2A2,B2,C2,D2). In the M0_GM group, which is phenotypically most similar to M1 macrophages and expresses the M1 marker CD86 [57], a significant reduction in TNFα and IL-6 mRNA expression was observed after incubation with exosomes (Figure 2A1,B1), but cytokine secretion by the cells remained unchanged (Figure 2C1,D1). MBV reduced IL-6 gene transcription in M0_M macrophages, but the cytokine concentration in the medium remained almost unchanged (Figure 2B3,D3). Overall, the results suggest that both types of UC-MSC EV—exosomes and MBV—possess strong anti-inflammatory activity against M1 macrophages, shifting their phenotype towards a lower pro-inflammatory cytokine secretion profile. Therefore, it can be concluded that UC-MSC MBV and exosomes have an approximately equal in vitro immunosuppressive effect regarding M1 macrophages by reducing the production of inflammatory cytokines characteristic of the M1 macrophage phenotype.

### 2.3. Impact of UC-MSC Exosomes and MBV on the Restoration of Articular Cartilage in Rats with OA

**Figure 3:**
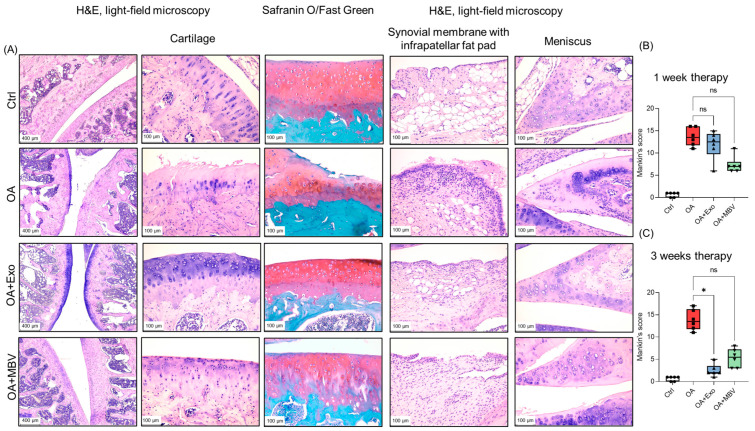
Histochemical analysis of the impact of EV (exosomes and MBV) from UC-MSC on the progression of pathological changes in articular tissues (cartilage, synovial membrane, infrapatellar fat pad, meniscus) in rats with surgically induced OA. (**A**) Histological staining with H&E, Safranin O/Fast Green. The images depict histological sections of animals 3 weeks post-OA therapy. Ctrl—intact control without surgery, OA—OA group injected intra-articularly with PBS, OA + Exo—group with intra-articular injection of EV from UC-MSC secretome, OA + MBV—group with intra-articular injection of MBV from UC-MSC. Scale bar: 100 µm. (**B**,**C**) Semi-quantitative evaluation of changes in cartilage tissue was conducted using the Mankin scoring method. B. One week after intra-articular injection of EV (exosomes and MBV) from UC-MSC. C. Three weeks after intra-articular injection of EV (exosomes and MBV) from UC-MSC. Experimental results are presented as boxplots showing the median ± minimum and maximum values. * *p* < 0.05; ns (*p* > 0.05). The figures (circle, square, triangle) on the graphs indicate the Mankin’s scores ● (control group); ■ (OA group); ▲ (experimental group + exosomes); ▼ (experimental group + MBV).

Three weeks after meniscectomy, articular cartilage samples with induced OA exhibited marked degenerative changes in joint tissues compared to the intact control. The number of viable chondrocytes in the articular cartilage decreased, with large acellular areas, cartilage thinning up to its complete disappearance, and replacement by fibrous tissue observed (Figure 3A, OA subgroup). The cartilage matrix stained with Safranin O showed reduced glycosaminoglycans (GAGs), resulting in greenish areas stained with Fast Green, with osteoporosis observed in some regions (Figure 3A, OA subgroup). The synovial membrane showed signs of proliferation and invaded the meniscal tissue. Additionally, increased fibrosis, localized synoviocyte proliferation, and pronounced lympho-macrophage infiltration were noted in the synovial membrane (Figure 3A, OA subgroup). In the infrapatellar fat pad (or Hoffa’s fat pad), lympho-macrophage infiltration, fibrosis, and increased vascularization were observed. In the cartilage tissue of the remaining meniscus, foci of dystrophically altered chondrocytes appeared, with synovial membrane invasion into the meniscal tissue and expanded ossification zones (Figure 3A, OA subgroup).

In both experimental groups treated with UC-MSC exosomes and MBV, after 3 weeks of therapy, the pathological condition of the joint improved, approaching normal (Figure 3A, OA + Exo, OA + MBV subgroups). The articular cartilage was preserved, had normal thickness and structure, and displayed orderly, evenly distributed chondrocytes with almost no signs of dystrophic changes (Figure 3A, OA + Exo subgroup). The cartilage matrix was mostly uniformly stained red, indicating high GAG content, corresponding to normal levels (Figure 3A, OA + Exo subgroup). After MBV treatment in animals with induced OA, the articular cartilage was largely preserved with a normal structure, although it occasionally appeared dystrophic and thinned in the superficial zone, with uneven chondrocyte distribution and acellular fields. Additionally, in the OA + MBV group, certain areas showed reduced GAGs, resulting in greenish staining with Fast Green, and osteoporosis was observed in some regions (Figure 3A, OA + MBV subgroup). After exosome and MBV therapy, cartilage tissue samples showed a focus on chondrogenesis, with chondrogenesis being more pronounced after exosome therapy (Figure 3A, OA + Exo, OA + MBV subgroups). The structures of the synovial membrane, infrapatellar fat pad and meniscus were close to normal, with significantly reduced lympho-macrophage infiltration, no synoviocyte proliferation, decreased fibrosis and vasculogenesis compared to the OA group (Figure 3A, OA + Exo, OA + MBV subgroups). Micrographs in the best resolution are presented in Supplementary Materials Figure S7.

An important distinction between the therapeutic effects of the two types of EV was observed after 1 week; MBV had a more pronounced therapeutic effect, showing a lower score for degenerative changes in articular cartilage using the Mankin method (Figure 3B). After 3 weeks of MBV therapy, the effect was more pronounced compared to the 1-week results, but the differences were not statistically significant compared to the OA subgroup (Figure 3C). The UC-MSC exosome group exhibited a less pronounced therapeutic effect than MBV after 1 week of treatment (Figure 3B). However, the best results were achieved in the exosome-treated group, where degenerative changes in the articular cartilage and lympho-macrophage infiltration were almost completely absent compared to the MBV-treated group (Figure 3C). These results suggest that UC-MSC exosomes and MBV significantly improve the morphological condition, with exosomes almost completely eliminating degenerative processes in the joints of rats with surgically induced OA, reaching maximal effect after 3 weeks of therapy.

### 2.4. UC-MSC Exosomes More Effectively Reduce Inflammation in Synovial and Cartilage Tissue in Rats with OA Compared to MBV from UC-MSC

**Figure 4:**
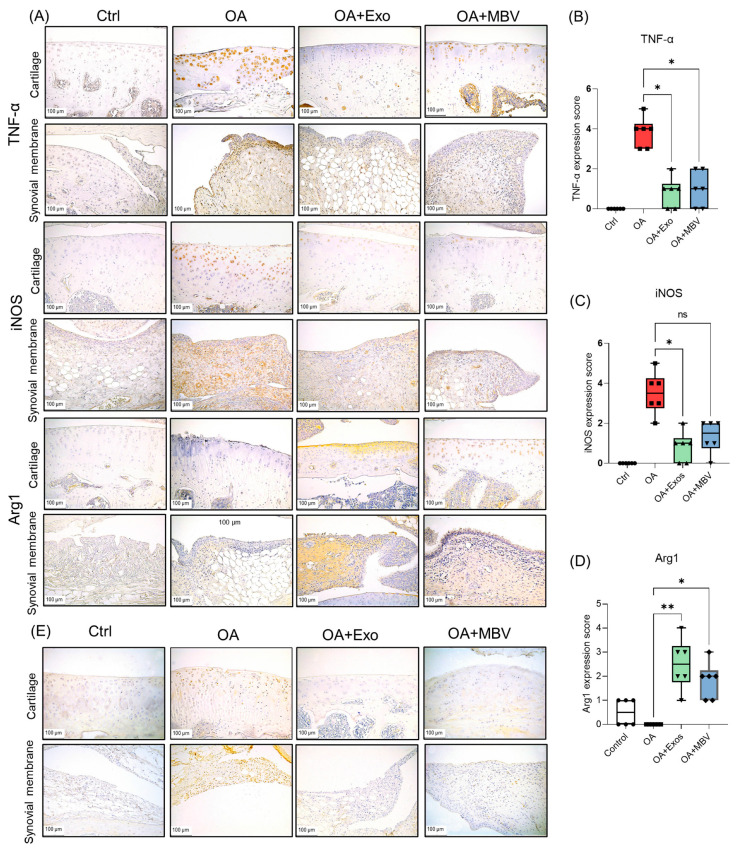
Immunohistochemical analysis of the impact of UC-MSC-derived EV (exosomes and MBV) on the development of inflammation in articular tissues of rats with surgically induced OA. (**A**) IHC analysis of inflammatory markers TNF-α and iNOS. Arg-1—marker of enhanced anti-inflammatory microenvironment. Histological tissue images were obtained 3 weeks post-OA therapy. Ctrl—intact control without surgery, OA—group with surgically induced OA injected with PBS, OA + Exo—group with intra-articular injection of UC-MSC exosomes, OA + MBV—group with intra-articular injection of UC-MSC MBV. (**B**–**D**) Semi-quantitative scoring of IHC marker expression in joint tissues across different groups. (**B**) TNF-α, (**C**) iNOS, (**D**) Arg-1. (**E**) in situ PCR analysis of NLRP3 inflammasome in joint tissue samples of experimental groups. Positively stained chondrocytes in the articular cartilage and macrophages in the synovial membrane and infrapatellar fat pad containing TNF-α, iNOS, Arg-1, and NLRP3 are shown in brown. * *p* < 0.05; ** *p* < 0.01; ns (*p* > 0.05). The figures (circle, square, triangle) on the graphs indicate the level of expression of IHC marker expression in joint tissues across different groups: ● (control group); ■ (OA group); ▲ (experimental group + exosomes); ▼ (experimental group + MBV).

Additionally, to analyze the impact of different types of EV on inflammatory processes in OA, we conducted an immunohistochemical (IHC) analysis of markers associated with OA-related inflammation. Markers linked to the inflammatory tissue microenvironment in OA, including TNF-α and inducible nitric oxide synthase (iNOS), were selected, along with arginase (Arg1), a marker associated with anti-inflammatory responses (Figure 4A). In the intact control group, almost no expression of TNF-α and iNOS was observed (Figure 4A). In the OA group without EV treatment, significant expression of these markers was observed in chondrocytes of dystrophic and degrading articular cartilage, as well as in numerous macrophages and fibroblasts in the synovial membrane and infrapatellar fat pad (Figure 4A–C). Unlike TNF-α, iNOS was also highly expressed in endothelial cells of the synovial membrane. High expression of these proteins indicated a pronounced inflammatory reaction in joint tissues in OA samples on day 42 post-meniscectomy for OA induction. Meanwhile, Arg1 was detected only in isolated macrophages of the synovial membrane and infrapatellar fat pad and a few chondrocytes in the articular cartilage of the intact control and was completely absent in the tissue samples from the OA group (Figure 4A,D). After three weeks following the injection of both types of UC-MSC EV, treatment with exosomes and MBV significantly reduced the expression of inflammatory markers TNF-α, to a lesser extent iNOS, and markedly increased Arg1 expression, indicating a strong anti-inflammatory effect of both types of EV (Figure 4A–D). The reduction in TNF-α and iNOS was predominantly observed in articular cartilage, synovial membrane and infrapatellar fat pad cells (Figure 4A). Strong Arg1 expression was primarily detected in the superficial zone of the articular cartilage and in macrophages infrapatellar fat pad (Figure 4A). Semi-quantitative evaluation of the expression of immunohistochemical markers demonstrated a statistically significant inhibitory effect of exosomes on TNF-α and iNOS expression levels (Figure 4B,C). MBV showed a weaker and statistically insignificant effect on the reduction in iNOS expression compared to TNF-α when compared with exosomes (Figure 4B,C). The impact of MBV and UC-MSC exosomes on Arg1 levels served as an indicator of a significant reduction in inflammation in the synovial and infrapatellar fat pad tissues due to the shift in macrophage polarization towards a phenotype with a more pronounced anti-inflammatory profile (Figure 4D). Micrographs and additional graphs of IHC results for TNF-α, iNOS and Arg-1 markers in individual tissues such as cartilage and infrapatellar fat pad have been added to Supplementary Materials Figures S8 and S9.

In addition to standard methods such as IHC, to assess the impact of UC-MSC EV on OA progression in vivo, we further evaluated the levels of NOD-like receptor 3 (NLRP3). This inflammasome serves as a marker of OA progression. We conducted a NLRP3 inflammasome analysis using PCR in situ, a method that allows for the amplification of DNA molecules within tissue cells, enabling gene localization [45]. Elevated levels of NLRP3 were observed in chondrocytes of the superficial layer of articular cartilage and in macrophages of the synovial membrane and infrapatellar fat pad in the OA group without treatment. In the intact control group, almost no NLRP3 expression was detected in the cartilage and synovial membrane and infrapatellar fat pad areas of the joint. In the group receiving Exo UC-MSC therapy, a reduction in NLRP3 levels was observed. MBV treatment also led to a decrease in NLRP3 amplification in the hyaline cartilage and synovial membrane areas of the joint. However, NLRP3 expression was detected in isolated fibroblasts and macrophages in the synovial membrane and infrapatellar fat pad, though the frequency of such cells was lower than in the untreated OA group (Figure 4E). These cumulative results demonstrate that UC-MSC exosomes offer a more effective treatment for OA compared to MBV from UC-MSC, positioning them as a promising source of EV for OA therapy.

## 3. Discussion

OA is a disease with complex etiology and pathogenesis. Currently, it is known that inflammation is one of the key features of OA, as components of the infrapatellar fat pad and the synovial membrane, fibroblast-like synoviocytes, and macrophages are pro-inflammatory activated in response to degrading ECM components of cartilage, as well as catabolically active chondrocytes secreting inflammatory factors IL-1β, TNF-α, and monocyte chemoattractant protein-1 [58,59]. In addition, the infrapatellar fat pad is an abundant source of adipokines and pro-inflammatory and catabolic cytokines, which may contribute to chronic synovial inflammation, cartilage destruction, and subchondral bone remodeling during knee osteoarthritis [8,60]. Activated pro-inflammatory factors M1-like macrophages secrete pro-inflammatory cytokines IL-1β, TNF-α, and IL-6, as well as MMPs, leading to enhanced ECM degradation and chondrocyte apoptosis [61]. One promising therapeutic approach is the ability of a therapeutic agent to alter the immune environment in joint tissues by promoting the reprogramming of macrophages from M1 to M2, a state with regenerative and immunoregulatory profiles due to the secretion of anti-inflammatory and trophic factors [62,63].

MSC and their secretome components, primarily EV, have shown great potential for the treatment of various degenerative diseases in which chronic inflammation is a key pathogenic factor [24,56,64]. EV have demonstrated a significant therapeutic effect in OA therapy in cellular and animal models, positively affecting the attenuation of the catabolic phenotype of chondrocytes and reducing ECM degradation processes of hyaline cartilage and subchondral bone, while exerting an anti-inflammatory effect by promoting the polarization of macrophages from M1 to M2 phenotype [65,66,67]. EV from bone marrow MSC inhibit inflammatory pathways such as NF-κB, p38, ERK1/2, PI3K, and TAK1 in different cell types involved in the pathogenesis of OA, such as chondrocytes and synoviocytes [68,69]. Adipose MSC-derived exosomes carrying miR-34 exhibit an inhibitory anti-inflammatory effect on macrophages by promoting M2 macrophage polarization through the suppression of NF-κB signaling, such as IRAK1 and TRAF6 [50,69,70]. The inhibition of the NF-κB signaling pathway is crucial in OA therapy, as increased expression of this nuclear factor induces the production of inflammatory cytokines IL-1β and proteases like MMPs, whose elevated activity in OA enhances ECM degradation [71]. In this study, we used UC-MSC to obtain exosomes and MBV, as this EV source has several advantages. UC-MSC have high proliferation rates, and exhibit pronounced immunosuppressive effects. Additionally, the umbilical cord as a source of MSC is relatively accessible [55,72,73]. EV from UC-MSC have rarely been studied for OA therapy compared to adipose and bone marrow MSC [64,74]. UC-MSC EV effectively suppressed inflammation by reducing NLRP3 levels in macrophages through the inhibition of METTL3 via exosomal miR-1208 [49].

In this study, we compared the therapeutic effects of EV from the UC-MSC secretome and the relatively understudied EV subtype—MBV. MBV is a component of the ECM of soft tissues, and they have demonstrated significant therapeutic potential due to their ability to stimulate the polarization of inflammatory M1 macrophages towards M2-like macrophages with immunosuppressive and regulatory profiles [43,44,75]. MBV is enriched with various microRNAs, such as miRNA125b-5p, 143–3p, and 45-5p, which mediate changes in M1 macrophage signaling pathways, promoting an increase in the number of M2 macrophages [44]. Additionally, the authors noted the important role of IL-33, identified in MBV derived from ECM of soft mucosal tissues, in regulating the immune phenotype of macrophages, providing regenerative effects in various diseases, such as skeletal muscle repair by modulating the anti-inflammatory activation of macrophages [75,76]. MBV has also shown promising results in rheumatoid arthritis (RA), an inflammatory condition with an autoimmune nature, unlike OA [77]. MBV demonstrated a therapeutic effect similar to methotrexate, a standard RA therapy, by increasing the population of M2-like CD43hi/His48lo/CD206+ macrophages and reducing the secretion of pro-inflammatory chemokines CXCL10 and MCP-3, levels of which were measured in the serum of rats with acute and chronic RA [77]. Most studies have used MBV obtained mainly from porcine urinary bladder and small intestine submucosa, while little attention has been paid to MBV derived from monolayer cell cultures [42,44]. For example, in the work by Hussey et al. [42], sequencing and lipidomic analyses of MBV and EV derived from the NIH-3T3 mouse fibroblast line were conducted. In their study, Hussey et al. demonstrated that MBV have a different profile miRNA and lipid composition from exosomes, indicating their distinct origin [42]. To date, only one study has been published that examines in detail the proteomic profile of MBV and exosomes derived from UC-MSC using mass spectrometry [41]. MBV and exosomes showed the presence of numerous immunoregulatory proteins, as well as proteins involved in ECM organization. However, in this study, IL-33 was not detected in MBV samples, a protein that has previously demonstrated a pronounced immunosuppressive effect of MBV on macrophage polarization [41,76]. These results suggest that EV from ECM cell cultures may be considered an alternative source of EV for OA therapy.

In this study, we compared the effects of exosomes and MBV derived from UC-MSC. Both types of EV demonstrated the ability to modulate the pro-inflammatory properties of MDMs, leading to a significant reduction in the production of pro-inflammatory cytokines IL-6 and TNF-α by M1 macrophages. Since the M1/M2 macrophage dichotomy does not fully reflect the diversity of macrophage polarization spectra, it can be assumed that both types of EV led to the formation of a population of macrophages with an intermediate phenotype between the two extreme points of the polarization continuum. However, in the therapy of OA in rats, UC-MSC MBV demonstrated a weaker therapeutic effect than UC-MSC exosomes. Therapy with UC-MSC exosomes resulted in a significant improvement in joint tissue condition, as shown by IHC. Exosomes led to the preservation of the hyaline cartilage ECM structure, which was closer to the intact control than MBV. Both types of EV; however, alleviated synovitis, reducing synoviocyte proliferation, and lympho-macrophage infiltration in the joints of rats with OA after EV therapy. MBV and exosomes showed different abilities to alter the levels of inflammatory cytokine and protein expression UC-MSC exosome therapy resulted in a more pronounced reduction in the expression of inflammatory factors TNF-α, iNOS, NLRP3, and increased expression of Arg-1 in macrophages and synovial fibroblasts compared to MBV. The results of the histological analysis using the Mankin’s method on joint tissue samples showed that both types of EV, exosomes and MBV, exhibited a strong anti-inflammatory effect. However, statistical analyses revealed that only exosomes (*p* = 0.0226) contributed to a statistically significant anatomical and morphological improvement in the knee joints of rats with induced OA. The differences in the therapeutic efficacy of MBV and EV derived from UC-MSC partially align with the results of our previous work, as the proteomic profile of exosomes showed a higher saturation with proteins related to ECM organization compared to MBV, which is an important factor in OA therapy [41]. In histological samples of joints in the group receiving exosomes, we observed better ECM condition in the hyaline cartilage compared to MBV. At a later stage of MBV therapy, more pronounced dystrophic processes in the hyaline cartilage were observed, accompanied by a decrease in GAG in some areas compared to the group receiving exosome treatment. One of the probable reasons for the differences in the therapeutic efficacy of MBV and exosomes from UC-MSC is the difference in pharmacokinetic parameters and the elimination rate of MBV and exosomes from the joint area. Additionally, future studies need to establish differences in the transcriptomic composition of both types of EV, as certain microRNAs may play a key role in regulating pathways related to the regeneration and inflammation modulation of both macrophages and other cells involved in OA development.

In this study, it is important to note several limitations. The research was conducted on a small sample size, with *n* = 12 per group. One significant limitation in studying MBV as an alternative EV source is the lack of specific markers that can reliably confirm the EV population. Furthermore, only one dosage of UC-MSC EV was used for both types, and there is insufficient data on how the increased frequency of injections or extended therapeutic duration might affect the histopathological structure of the knee joints in OA rats. Another limiting factor is the study’s focus on inflammation regulation in OA, while other cellular models also consider changes in ECM metabolism, chondrocyte survival, and proliferation. Future research should further explore the reasons for the different therapeutic effects of MBV and exosomes derived from UC-MSC, as well as investigate the influence of EV from other MSC sources, such as adipose tissue and bone marrow MSC.

## 4. Materials and Methods

### 4.1. Culturing of UC-MSC

Human UC-MSC were obtained from the Biobank of the Institute of Regenerative Medicine (Sechenov University, Moscow, Russia). UC-MSC were isolated from Wharton’s jelly of the human umbilical cord. In accordance with the Declaration of Helsinki, the sample was collected after the donor signed an informed consent form approved by the local ethics committee of Sechenov University (No. 16–21, 16 September 2021, Moscow, Russia). UC-MSC were isolated using a protocol described by Peshkova et al. [78] with minor modifications.

Cells were cultured at 37 °C in a 5% CO_2_ atmosphere. Cells were cultured in a complete growth medium composed of DMEM/F12 (1:1) (Gibco, Norristown, PA, USA) supplemented with 10% fetal bovine serum FBS (HyClone, Logan, UT, USA). L-glutamine 2 mM (Biolot, Novosibirsk Oblast, Russia), gentamicin 50 µg/mL (PanEco, Moscow, Russia), insulin-transferrin-selenium 1:100 (Biolot, Russia), bFGF 20 ng/mL (Prospec, Rehovot, Israel). The medium was replaced every 2–3 days. Cells no older than the 4th passage were used for immunophenotype characterization and EV isolation. Cells underwent no passaging for 14 days prior to EV isolation for better ECM accumulation. Forty-eight hours prior to EV isolation, the complete growth medium was removed, and the Petri dishes were washed three times with Hank’s Balanced Salt Solution (HBSS) to remove serum residues. Cells were then left in serum-free DMEM/F12 supplemented with 2 mM L-glutamine (Biolot, Russia)

### 4.2. Immunophenotype Analysis of UC-MSC

UC-MSC p4 were treated with Versene solution (Invitrogen, Waltham, MA, USA) and 0.25% trypsin solution (Invitrogen, USA) to obtain single-cell suspensions. The resulting cell suspensions, containing at least 1 million cells each, were further washed with phosphate-buffered saline (PBS) to remove any remaining culture medium. The obtained UC-MSC suspensions were stained with anti-human antibodies to CD105, CD73, CD90, CD44, CD19, CD11beta, CD45, and CD34 (hMSC Analysis Kit, BD Stemflow™, San Diego, CA, USA). Staining was performed according to the manufacturer’s protocol. Cells stained using isotype control from the kit (BD Stemflow™ hMSC Analysis Kit) and unstained cell suspensions were used as controls. After a 15 min incubation, the samples were washed with PBS and analyzed using a Sony SH800 cell sorter (Sony Biotechnology, San Jose, CA, USA). Background fluorescence levels were determined using unstained cell suspensions, while specificity for antibodies was verified by comparing unstained suspensions with isotype controls. Each marker was then compared to the respective isotype control.

### 4.3. Isolation of MBV from ECM and Exosomes from the Conditioned Medium of UC-MSC

UC-MSC were cultured in Petri dishes for 14 days for sufficient synthesis of the extracellular matrix. The conditioned medium was removed from the dishes for isolation exosomes, and UC-MSC were washed three times with PBS solution. ECM proteolysis for isolation MBV was performed by adding 1 mL of the following enzyme solution: dispase (1.5 U/mL, Gibco™, 17105041, Grand Island, NY, USA), collagenase I (2 mg/mL, Gibco™, 17100017), and collagenase II (1 mg/mL, Gibco™, 17101015) into each dish. The dishes were incubated at 37 °C for 60 min. After proteolysis, the contents of the dishes and the UC-MSC conditioned medium (CM) samples were transferred into centrifuge tubes and centrifuged at 400× *g* for 10 min and at 2500× *g* for 20 min. The isolation of exosomes from CM was carried out in parallel with MBV in a similar mode to MBV isolation (at 400× *g* for 10 min and at 2500× *g* for 20 min). The supernatant samples with exosomes and MBV were transferred to clean ultracentrifuge tubes and centrifuged at 10,000× *g* for 30 min. The collected supernatant exosomes and MBV were passed through a syringe filter with a 0.22 µm pore size. Ultracentrifugation was performed twice at 120,000× *g* for 90 min at 4 °C (Optima XPN-100, Beckman Coulter, Indianapolis, IN, USA). After ultracentrifugation, the supernatant was discarded, and the pellet was resuspended in sterile PBS and stored at −80 °C.

### 4.4. Evaluation of the Morphology and Size of UC-MSC Extracellular Vesicles

To characterize the number of vesicles obtained, the protein content was measured using the QuantiPro™ BCA Assay Kit (Sigma-Aldrich Co. LLC, Burlington, MA, USA). The protein concentration in the samples was measured using the Pierce™ BCA Protein Assay Kit (Thermo Scientific, Norristown, PA, USA), and all samples were diluted with PBS to a final protein concentration of approximately 150 µg/mL. For the analysis of EV morphology using transmission electron microscopy (TEM), the EV samples were placed on carbon-coated TEM grids (Ted Pella, Redding, CA, USA). The vesicles were applied to the grids for 3 min, contrasted twice with uranyl acetate (1% aqueous solution), and air-dried. The morphology of EV was examined using a JEM-1011 transmission electron microscope (Jeol, Tokyo, Japan). The average size of ECM-derived MBV and conditioned medium EV from the UC-MSC secretome was determined using the Zetasizer Nano ZS instrument (Malvern Panalytical, Malvern, UK) as described in the methodology from [41].

### 4.5. Analysis of Exosomal Markers in MBV and EV from UC-MSC by Western Blot

To confirm that the isolated vesicles from CM are exosomes and MBV, an analysis of exosomal markers was performed by Western blotting. For this, the samples were treated with 4× Laemmli buffer and subjected to electrophoresis in a 12% polyacrylamide gel using a vertical chamber (BioRad, Hercules, CA, USA). Fifteen micrograms of protein were loaded into each lane. The transfer of proteins to a 0.2 µm nitrocellulose membrane (BioRad, USA) was carried out using a Power Blotter semi-dry blotting system (Invitrogen, USA) and the Power Blotter 10-step Transfer buffer (Invitrogen, USA). Then, the membrane was washed with Tris-buffered saline with 0.1% Tween (TBST) and incubated in blocking solution (5% BSA on TBST) for 1 h. Then, the membrane was incubated over night with primary antibodies against exosomal markers HSP70 dilution 1:1000 (Abcam, ab181601, Waltham, MA, USA), and TSG101 dilution 1:5000 (Abcam, ab125011), washed with TBST and incubated with secondary HRP-conjugated antibodies 1:10,000 (Arigobio, Hangzhou, China). The protein bands were detected using the ECL kit (Thermo Fisher, Waltham, MA, USA). Signal detection was performed using the iBright CL1500 imaging system (Invitrogen, USA).

### 4.6. Staining of MBV and Exosomes from UC-MSC with the PKH26 Membrane Dye

Freshly isolated MBV and exosomes from UC-MSC were stained with PKH26 according to the manufacturer’s instructions (Lumiprobe, Moscow, Russia). In the next step, the sample was resuspended, and 2 mL of 10% BSA was added to the EV, adjusting the volume to 8.5 mL. To the bottom of the ultracentrifuge tube, 1.5 mL of a 0.971 M sucrose solution was added without causing turbulence. Ultracentrifugation was performed at 190,000× *g* for 2 h at 2–8 °C. After ultracentrifugation, the top layer was collected, and the pellet was transferred to an Amicon MWCO (molecular weight cutoff) filtration column with a molecular weight of 10 kDa. Then, 9 mL of PBS and 0.75 mL of medium were added to remove the remaining impurities of the dye not bound to the EV. The sample was then centrifuged at 3000× *g* in a high-speed centrifuge for 40 min. For the visualization of MBV and exosomes on a confocal microscope, 10 µL of the stained vesicle suspension was applied to a poly L-lysine-coated adhesive slide and air-dried for 30 min in the dark. Next, the sample was fixed using ImmunoHistoMount Medium (Abcam), covered with a coverslip, and examined under an Olympus FV3000 laser scanning confocal microscope (Japan) at 100× magnification using immersion.

### 4.7. Culturing Macrophages Derived from Peripheral Blood Mononuclear Cells (PBMC)

**Figure 5:**
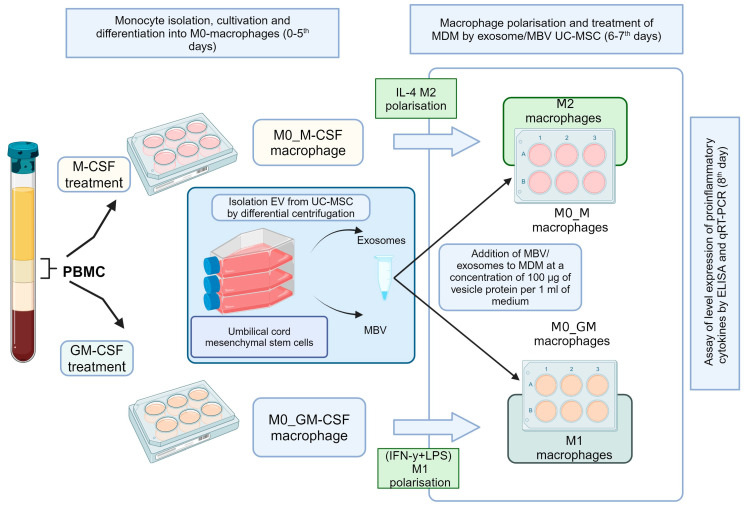
Scheme of the experiments with MDM and UC-MSC EV.

Monocytes were isolated from PBMC and obtained from healthy volunteers by age 18–60 years, with a body mass index of 18.5–24.9. All donors signed an informed consent form approved by the local ethics committee of Sechenov University (No. 16–21, 16 September 2021, Moscow, Russia). The isolation of PBMC and the culturing of monocyte-derived macrophages (MDM) were performed according to the standard protocols we described previously [57]. Macrophages were cultured in medium containing 50 ng/mL of GM-CSF or 50 ng/mL of M-CSF (SCI-store, Moscow, Russia), with medium changes on day 3. On day 6, the medium was changed again, leaving part of the MDM for both growth factors unpolarized as M0_GM and M0_M. For M1 macrophage polarization, LPS 10 ng/mL (Thermo Fisher Scientific, Carlsbad, CA, USA) and IFN-γ 50 ng/mL (Thermo Fisher Scientific, USA) were added; for M2 polarization, IL-4 25 ng/mL (SCI-store, Moscow, Russia) was added. MBV or exosomes from UC-MSC were then added to the cells at a concentration of 100 µg of vesicle protein per 1 mL of medium, while no additives were made to the control samples. MDMs were cultured with EV and polarization inducers for 48 h, after which the medium was collected for cytokine measurements, and cells were lysed to extract RNA. A detailed Scheme of the experiments with MDM and UC-MSC EV can be seen in Figure 5. To measure the expression and secretion levels of pro-inflammatory cytokines using qRT-PCR and ELISA, respectively, at least 6 experiments were conducted with 3 technical replicates for each sample.

### 4.8. Phagocytosis of MDM of MBV or UC-MSC Exosomes Labeled with Membrane Dye PKH26

Labeled PKH26 EV (MBV or UC-MSC exosomes) isolated by ultracentrifugation followed by ultrafiltration were added to pro-inflammatory macrophages M1-GM (IFN-y + LPS) at a concentration of 100 μg of vesicle protein per 1 mL of medium. The macrophages were then cultured for 24 h at 37 °C. The following day, the preparation was fixed with 4% paraformaldehyde, permeabilized with 1% TritonX-100, and the cell nuclei were stained with DAPI (2–2.5 μg/mL). The samples were then analyzed using an Olympus FV3000 confocal microscope (Japan) with 488 and 568 nm wavelength lasers for excitation. PKH26 exhibits red fluorescence (Ex 581/Em591), while DAPI has peak fluorescence in the blue range (Ex 358/Em 461).

### 4.9. Quantitative Polymerase Chain Reaction with Reverse Transcription and ELISA

Total RNA was extracted from cell lysates (approximately 280,000–320,000 macrophages per sample) using the RUplus-250 column-based RNA extraction kit (Biolabmix, Russia). RNA quality was assessed using a Nanodrop 8000 (Thermo Scientific, USA). For reverse transcription from RNA to cDNA, 1 μg of RNA per reaction was used with MMLV reverse transcriptase in the MMLV RT kit (Evrogen, Moscow, Russia). Quantitative reverse transcription PCR (qRT-PCR) was then performed using a ready-made 5X qPCRmix-HS SYBR mixture (Evrogen, Russia), using 50 μg of complementary DNA per reaction. Primer sequences were obtained from Primer-BLAST (https://www.ncbi.nlm.nih.gov/tools/primer-blast/) (accessed on 28 March 2024). Primer sequences used in the study were: GAPDH_forward GCACCGTCAAGGCTGAGAAC, GAPDH_reverse CCACTTGATTTTGGAGGGATCT; TNF_forward CTCTTCTGCCTGCTGCACTTTG, TNF_reverse ATGGGCTACAGGCTTGTCACTC; IL-6 _forward GGCACTGGCAGAAAACAACC, IL-6 _reverse CACCAGGCAAGTCTCCTCAT. PCR conditions: initial denaturation at 95 °C for 3 min, followed by 40 cycles of denaturation at 95 °C for 15 s, annealing at 60 °C for 15 s, and elongation at 70 °C for 20 s. Relative gene expression was analyzed using the ΔΔCt method, normalized to GAPDH (*n* = 2^−ΔC × 1000^, where ΔC = C1 − Chk, with C1 being the target gene and Chk the reference gene GAPDH).

On the eighth day of culturing, supernatants were collected from MDM and centrifuged at 400× *g* for 10 min to exclude cell debris. The concentrations of cytokines characteristic of pro-inflammatory macrophages, TNF-α and IL-6, were measured using an ELISA kit (Cytokine, St. Petersburg, Russia) according to the manufacturer’s instructions. Optical density was measured using a Multiskan™ FC Microplate Photometer (ThermoFisher, USA).

### 4.10. Induction of an OA Animal Model and Administration of EV

The experiment on 12–14 weeks-old Wistar rats (males, 200–250 g) was approved by the Local Ethics Committee of Sechenov University (protocol meeting No. 10–24 from 18 April 2024). The animals were housed in three per cage in standard conditions with a natural light cycle and free access to food and water. Before the operation to induce OA, the rats were anesthetized by intramuscular injection of ZOLETIL 100 (VIRBAC, Carros, France) at a dose of 6 mg of active ingredient per 1 kg of body weight. Of the 42 laboratory animals, a randomly selected group served as healthy controls (*n* = 6), while the remaining animals (*n* = 36) underwent. Meniscectomy to model OA as described by Yanagisawa et al. [79]. On the 21st day after induction of the OA model by meniscectomy experiment, the model animals were randomly divided into 4 subgroups: the first subgroup (*n* = 12) received an intra-articular injection of MBV (OA + MBV); the second subgroup (*n* = 12) received an intra-articular injection of exosomes (OA + Exo); the third subgroup (*n* = 12) received an intra-articular injection of PBS (OA + PBS, placebo). The dosage of MBV and EV from UC-MSC used in the study was 300 μg/mL of protein. The volume of the intra-articular injection was 20 μL of EV solution or sterile phosphate buffer. The animals (n=6) to each experimental subgroup and (n=3) in healthy control group were euthanized 7 and 21 days after the injections (the day the experiment ended). Animals were euthanized in a CO_2_ gas chamber for rodents’ sacrifice (AwTech, Moscow, Russia), where they received a 30% CO_2_ air flow in the first stage and 70% CO_2_ air flow in the second stage. Total euthanasia time was 10 min [80].

### 4.11. Histological Staining of Samples

All knee joint samples were fixed in 10% buffered neutral formalin, decalcified, dehydrated, embedded in paraffin, cut into 4 μm thick sections, and stained with hematoxylin-eosin and Safranin-O/Fast Green. The samples were examined using light-field and phase-contrast microscopy on a Leica DM 4000 B LED microscope with a Leica DFC 7000 T camera, operated with LAS V4.8 software (Leica Microsystems, Wetzlar, Germany). Morphological signs of cartilage tissue destruction and dystrophy in each knee joint sample were evaluated using the Mankin’s method in 5 different fields of view at 200× magnification [81] (see Table S1 in Supplementary Materials).

### 4.12. Immunohistochemistry

Cartilage tissues were collected and subjected to a histological analysis. The collected samples were fixed in 10% formalin, decalcified with 10% EDTA for 28 days, dehydrated, embedded in paraffin, and sectioned into 4 μm thick slices. To prepare samples for IHC, the sections were deparaffinized in xylene and isopropanol solutions. Antigen retrieval was performed at 70 °C for 30 min in retrieval buffer (Antigen retrieval buffer 100× Tris-EDTA, pH 9) (ab93684, Abcam). Endogenous peroxidase activity was blocked using a 3% hydrogen peroxide solution, and nonspecific protein binding was blocked with 10% Goat serum (Sigma 31872). Incubation with primary antibodies was performed for 30 min at room temperature at the following dilutions: 1:200 anti-ARG-1 (MAB120Ra21), 1:200 anti-TNFa (MAA133Ra21), 1:100 anti-NOS2 (PAA837Ra02) (Cloud-clone Corp., Wuhan, China). Secondary antibodies conjugated with horseradish peroxidase were incubated for 30 min at room temperature at the following dilutions: 1:1000 Goat anti-Mouse IgG (for anti-TNF, anti-Arg-1) (G-21040, Invitrogen), 1:20,000 Goat Anti-Rabbit IgG (for anti-NOS2) (205718, Abcam). Samples were stained using a 3,3′-diaminobenzidine tetrahydrochloride substrate mixture, followed by counterstaining with hematoxylin. Immunohistochemistry results were assessed semi-quantitatively in 5 different fields of view at 400× magnification using a scoring system (see Table S2 in Supplementary Materials).

### 4.13. In Situ Polymerase Chain Reaction

Tissue samples were prepared for in situ polymerase chain reaction in a similar way to the samples for IHC. After antigen retrieval, the samples were treated with 2 mg/mL pepsin in 10 mM HCL solution. Genomic DNA amplification was performed on an in situ PCR amplifier (Eppendorf, Leipzig, Germany). Primers for NLRP-3 were used: forward primer 5′-3′: AGCAGCAGGCATCGGAAAAACAAT, reverse primer 5′-3′: ACGTGGCGGGGGTGGTC. Primer concentration was 1 μM. The amplification mixture contained Biotin-11-dUTP 1/3 (Sileks, Moscow, Russia) and Taq polymerase in a 10× buffer (Evrogen, Russia). Amplification conditions: 30 cycles (Denaturation 95 °C for 2 min, Denaturation 95 °C for 15 s, Annealing 60 °C for 15 s, Elongation 72 °C for 30 s). After amplification, endogenous peroxidase was blocked with 3% hydrogen peroxide solution, and nonspecific binding sites were blocked with 10% Goat serum (Sigma 31872). Secondary antibodies against biotin, labeled with streptavidin-peroxidase, were applied at a 1:200 dilution (21132, ThermoScientific, USA). Samples were stained using a 3,3′-diaminobenzidine tetrahydrochloride substrate mixture, followed by counterstaining with hematoxylin. Samples were examined using light-field and phase-contrast microscopy on a Leica DM 4000 B LED microscope with a Leica DFC 7000 T camera, operated with LAS V.

### 4.14. Statistical Analysis

Statistical analyses of experimental data were performed using GraphPadPrism 10.00 software for Windows (GraphPad Software, San Diego, CA, USA). The normality of the distribution was determined using the Shapiro–Wilk test. The Kruskal–Wallis test followed by Dunn’s post hoc test was used to assess intergroup differences. *p*-values ≤ 0.05 were considered statistically significant. The results of the statistical analyses were presented as a box plot indicating the median values of the variable.

## 5. Conclusions

The results of this study showed that EV and MBV derived from UC-MSC can exert a therapeutic effect on OA progression in rats. In an experimental OA model, MBV showed better therapeutic effects in the early stages, within the first week of OA therapy. However, by the end of the 3 week therapy, exosomes demonstrated better therapeutic properties, contributing to improved histological characteristics in the joint cartilage tissue and the preservation of the hyaline cartilage ECM structure with minimal signs of dystrophic processes. Additionally, UC-MSC exosomes showed a superior ability to mitigate inflammatory processes in cartilage tissue and the synovial membrane compared to MBV, significantly reducing the expression of TNF-α and iNOS while increasing Arg-1. UC-MSC MBV and exosomes had pronounced immunosuppressive effects on human MDMs, reducing mRNA expression and cytokine secretion of IL-6 and TNF-α in pro-inflammatory M1 macrophages, which is crucial for reducing the degree of inflammation, including in OA. The obtained results allow us to consider exosomes from UC-MSC as a more effective and promising therapeutic agent for OA treatment.

## Data Availability

Data generated during the study and included in this article are available from the corresponding authors upon request.

## Supplementary Materials

The following supporting information can be downloaded at: https://www.mdpi.com/article/10.3390/ijms252111564/s1.

## Abbreviations

ARG-1: arginase-1; CXCL10, C-X-C motif chemokine ligand 10; CD, cluster of differentiation; DAMP, damage-associated molecular patterns; ECM, extracellular matrix; ELISA; enzyme-linked immunosorbent assay; EV, extracellular vesicles; ECM, extracellular matrix; GM-CSF, granulocyte-macrophage colony-stimulating factor; HSP70, 70 kilodalton heat shock protein; IFN-γ, Interferon-γ; IHC, immunohistochemistry; iNOS, inducible nitric oxide synthase; IL, interleukin; LPS, lipopolysaccharide; M-CSF, macrophage colony-stimulating factor; MBV, matrix-bound nanovesicle; MIA, monoiodoacetate; MMx, medial meniscectomy; MSC, mesenchymal stromal cells; MDM, monocyte derived macrophages; MMPs, matrix metalloproteinase; miR, microRNA; NF-κB, nuclear factor kappa-light-chain-enhancer of activated B cells; NLRP3, NLR family pyrin domain containing 3; OA, osteoarthritis; PBMC, peripheral blood mononuclear cell; PBS, phosphate-buffered saline; qRT-PCR, quantitative Reverse transcription polymerase chain reaction; TEM, transmission electron microscopy; TNF, tumor necrosis factor; TSG101, tumor susceptibility gene 101; UC-MSC, umbilical cord mesenchymal stromal cells.
