# Supplementary material for: The Therapeutic Potential of Exosomes vs. Matrix-Bound Nanovesicles from Human Umbilical Cord Mesenchymal Stromal Cells in Osteoarthritis Treatment
Source: Int J Mol Sci. 2024 Oct 28;25(21):11564. doi: 10.3390/ijms252111564 (PMC11545893; doi:10.3390/ijms252111564)
Supplement: Supplementary file 1 [file ijms-25-11564-s001.zip › ijms-3234963-supplementary.pdf]

Supplementary Material for

The therapeutic potential of exosomes vs. matrix-bound nanovesicles from human umbilical cord mesenchymal stromal cells in osteoarthritis treatment

Timofey O. Klyucherev[1,2], Maria A. Peshkova[1], Daria P. Revokatova[1,2], Natalia B. Serejnikova[1,3], Nafisa M. Fayzullina[1], Alexey L. Fayzullin[1,3], Boris P. Ershov[1], Yana I. Khristidis[1], Irina I. Vlasova[1], Nastasia V. Kosheleva[1,2], Andrey A. Svistunov[5] and Peter S. Timashev[1,2,4]

[1] Institute for Regenerative Medicine, I. M. Sechenov First Moscow State Medical University, Moscow, 119991, Russia;

[2] Laboratory of Clinical Smart Nanotechnologies, Institute for Regenerative Medicine, Sechenov University, 119991 Moscow, Russia;

[3] Laboratory of Digital Microscopic Analysis, Institute for Regenerative Medicine, Sechenov University, 119991 Moscow, Russia;

[4] World-Class Research Center "Digital biodesign and personalized healthcare", I. M. Sechenov First Moscow State Medical University, Moscow, 119991, Russia

[5]Sechenov First Moscow State Medical University, Moscow, Russia, 119991.

Corresponding author: Prof. Peter Timashev timashev_p_s@staff.sechenov.ru

Figure S1. Characterization of UC-MSC Exosomes, and UC-MSC MBV. (A) Flow cytometry results show the presence of phenotypic markers for UC-MSC (CD105, CD73, CD90, CD29, CD44) and the absence of hematopoietic markers (CD19, CD11beta, CD45, CD34).

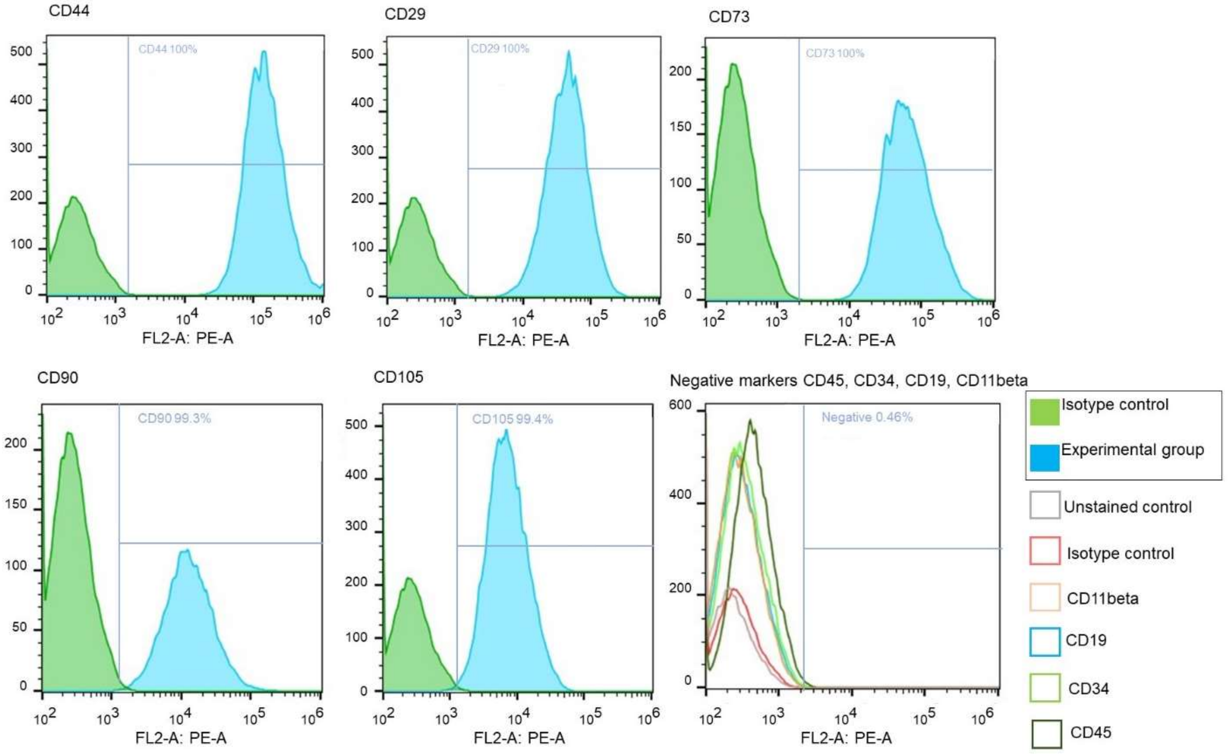

Figure S2. Particle size distribution obtained by nanoparticle dynamic light scattering.

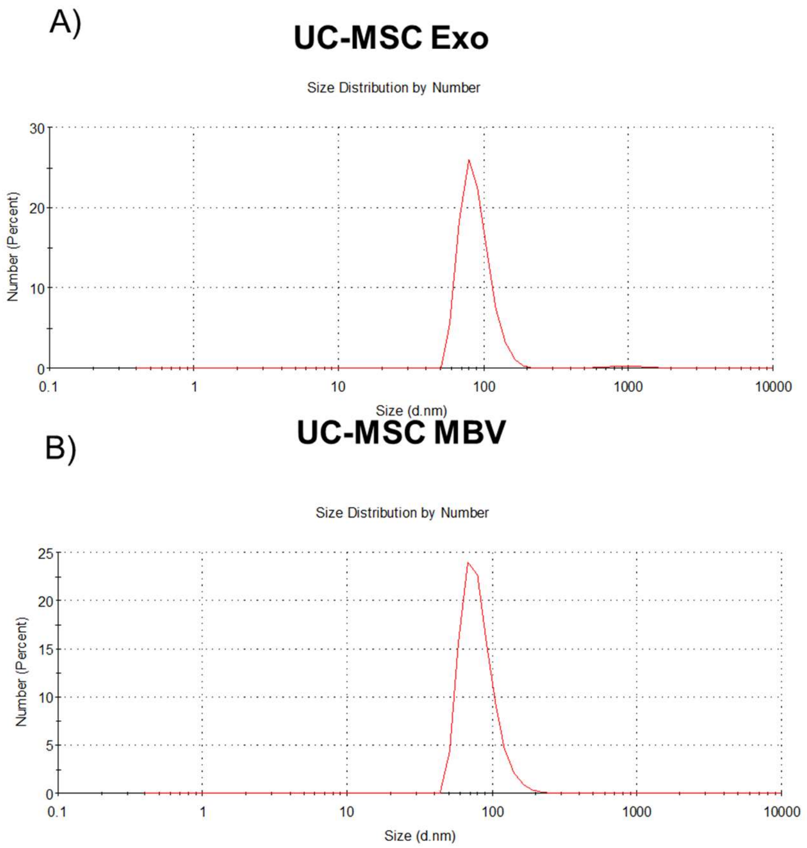

The histogram shows the peaks of particle size distribution for A) UC-MSC Exosomes, B) UC-MSC MBV. The Y-axis shows the distribution of number Particle size distribution (PSD) is the means (percentage), the X-axis shows the distribution of particle Size (d. nm)

Figure S3Visualization of UC-MSC Exo and UC-MSC MBV via transmission electron microscopy

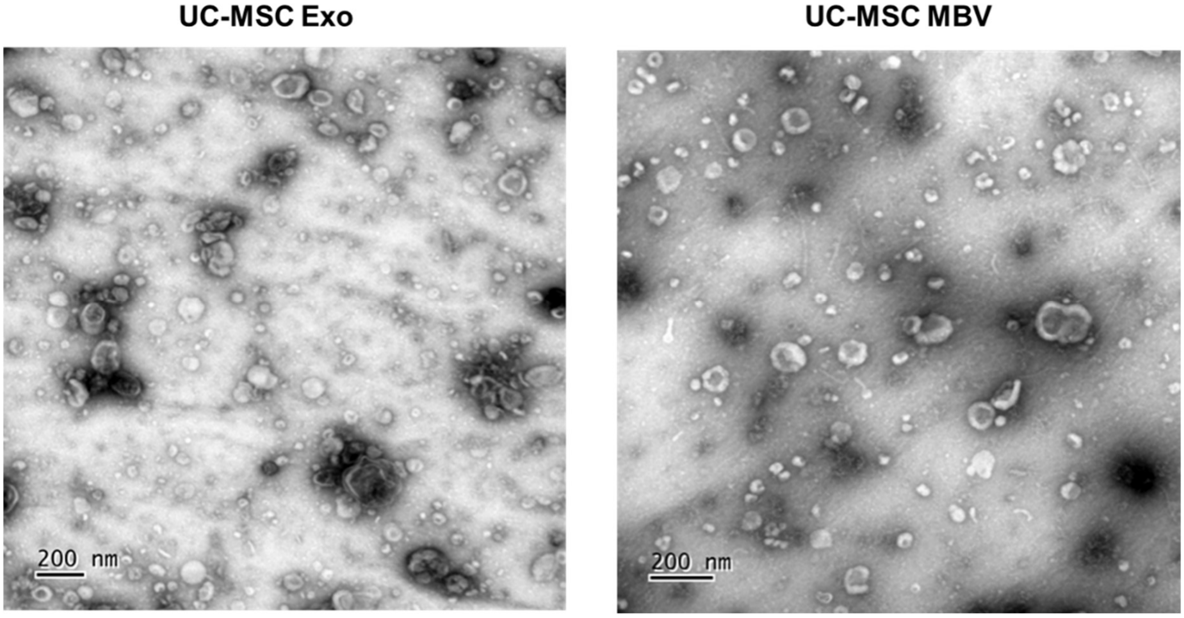

Figure S4. Visualization of UC-MSC Exo and UC-MSC MBV via confocal microscopy with fluorescent staining of extracellular vesicles (UC-MSC Exosomes and UC-MSC MBV) using PKH26 membrane dye.

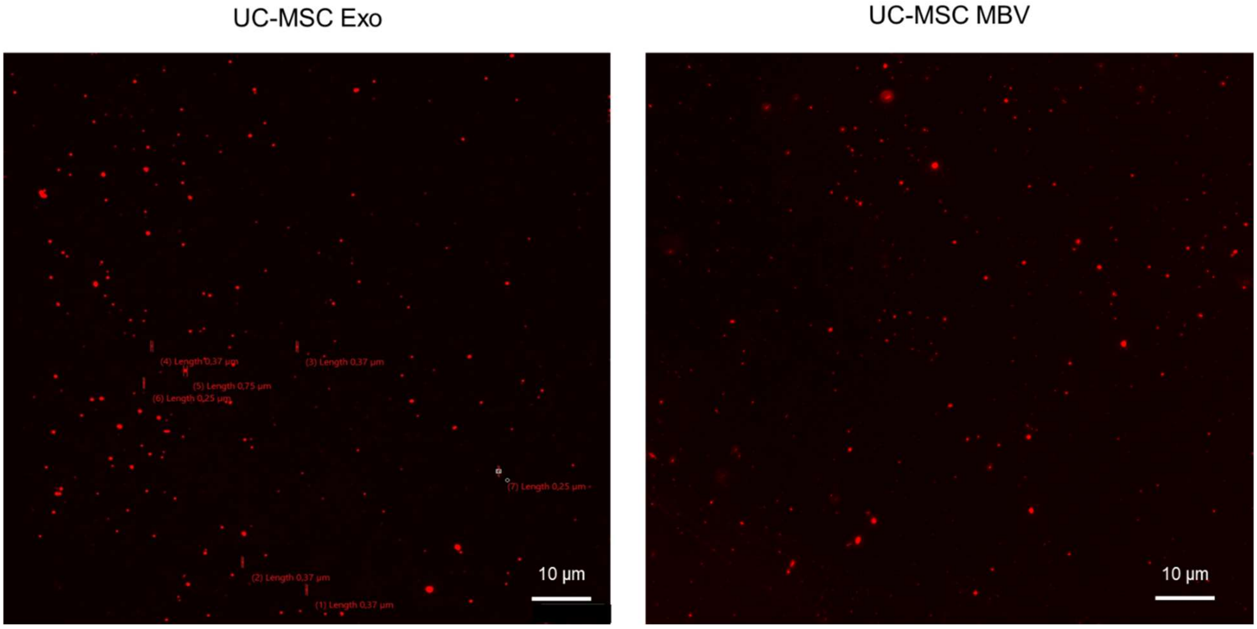

Figure S5. Phagocytosis of UC-MSC Exo and UC-MSC MBV labeled with PKH26 by M1 (IFN-γ+LPS) macrophages.

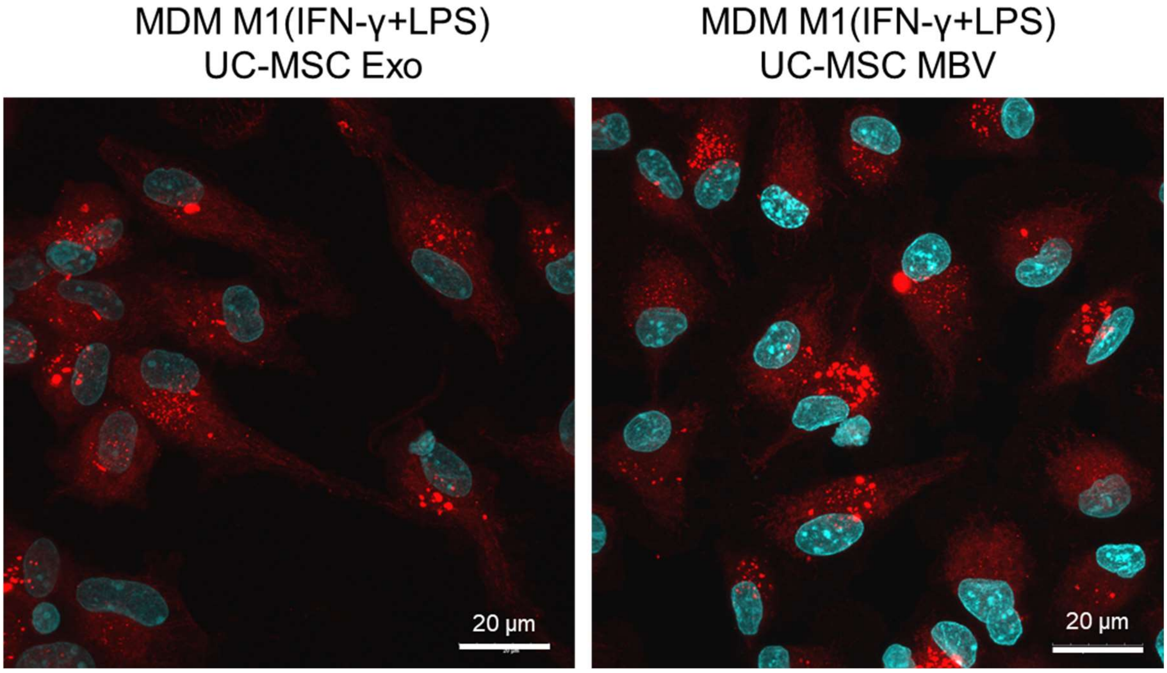

Figure S6. Flow cytometry assessment of MDM polarization markers.

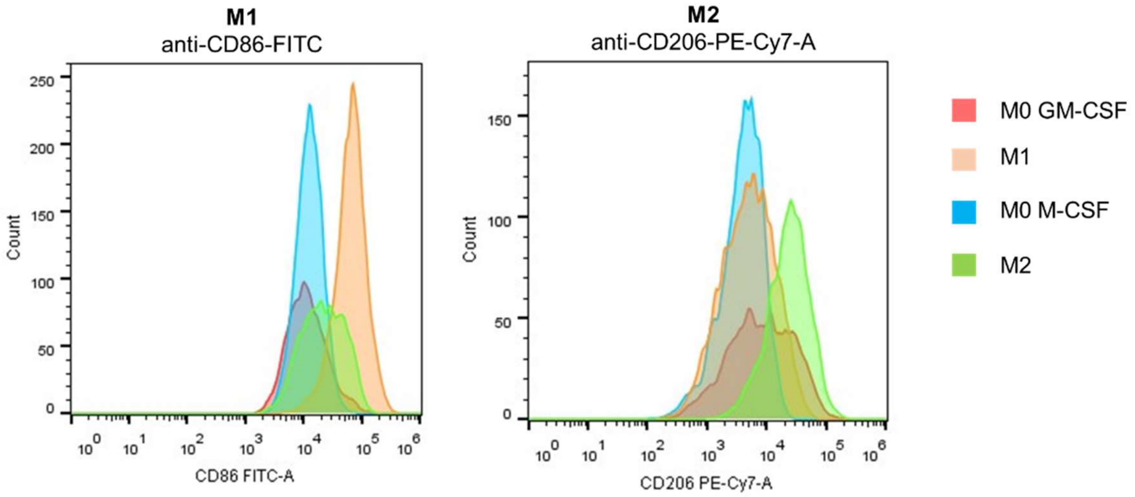

A. The histogram shows the peaks of fluorescence intensity for 4 MDM phenotypes used in the study (M0_GM, M1, M0_M, M2). Cells M0_GM, M1 were cultured with GM-SCF (50 ng/ml) for 6 days, on day 6 M1 were treated with LPS (10 ng/mlml) and IFN-γ (50 ng/ml). Macrophages of phenotypes M0_M, M2 were cultured with M-CSF (50 ng/ml) for 6 days, on the 6th day M2 were treated with IL-4 (10 ng/ml). After two days of culture with polarization inducers, macrophages were detached from the plastic surface using accutase after pretreatment with a Versene solution, after which the cells were stained with anti-CD86-FITC and/or CD206-PE-Cy7 antibodies.

Table S1. Semi-quantitative scoring of cartilage tissue changes using the Mankin method.

Features		Points
SurfaceIntegrity	Normal	0
	Minor surface changes (thickness variation)	1
	Moderate surface changes (thickness variation)	2
	Pronounced surface changes (thickness variation)	3
	Cracks in the superficial zone	4
	Cracks in the transitional zone	5
	Cracks extending to the calcified zone	6
	Fibrillation and/or absence of the superficial zone	7
	Fibrillation and/or absence of the transitional zone	8
	Fibrillation and/or absence of the calcified zone	9
	Fibrillation and/or absence of subchondral bone	10
CellularDystrophy	Normal	0
	Mild dystrophy	1
	Moderate dystrophy	2
	Severe dystrophy	3
	No cells	4
Cell Cloning	Normal	0
	Individual doublets	1
	Multiple doublets	2
	Doublets and triplets	3
	Multiple "nests" of cells	4

Maximumtotalscore: 18 points

Table S2. *Semi-quantitative scoring scale for immunohistochemical analysis data*

**PrevalenceofStaining**	**StainingIntensity**
0 – lessthan 25% ofcells	0 – notdetected
1 – 25-49% ofcells	1 – singlepositivelystainedgranules
2 – 50-74% ofcells	2 – moderate content of positively stained granules
3 – 75% ofcellsormore	3 – highcontentofgranules

Figure S7.

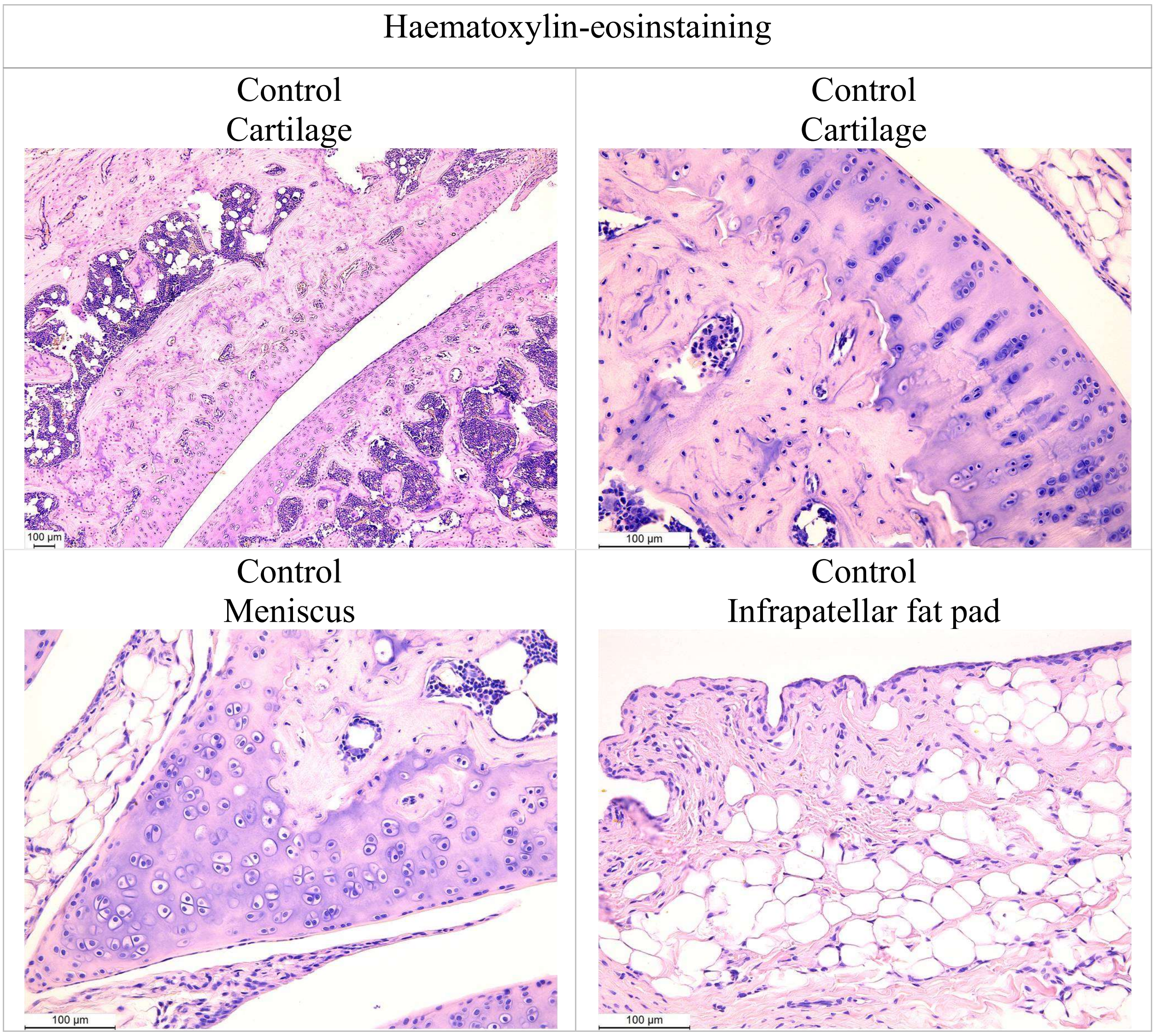

OA<br>Cartilage<br>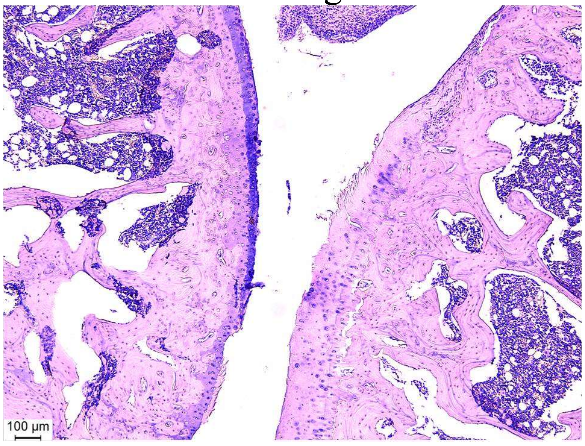 	OA<br>Cartilage<br>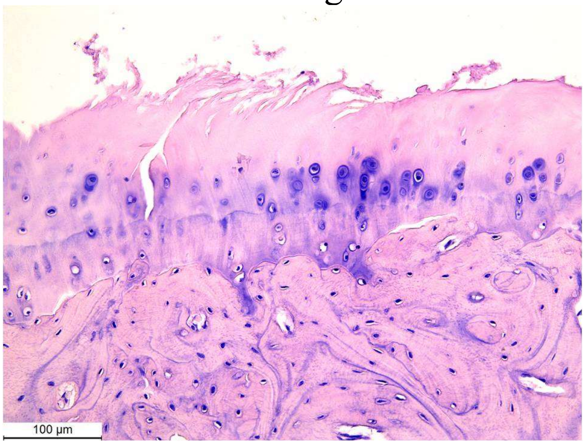
OA<br>Meniscus<br>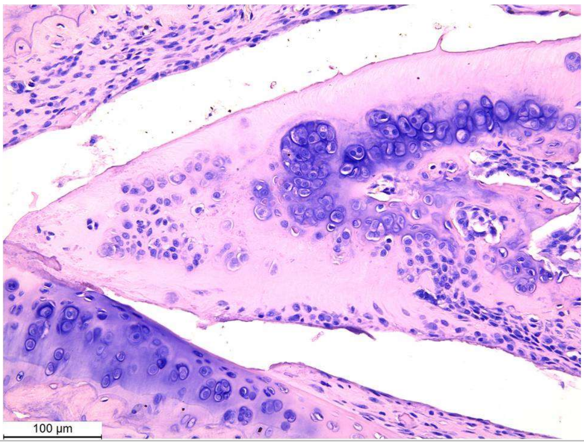 	OA<br>Infrapatellar fat pad<br>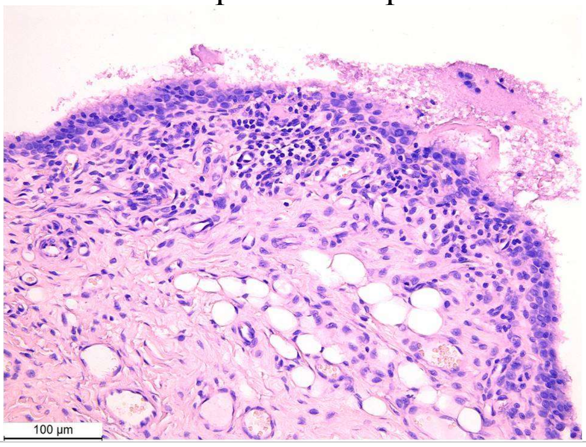
OA+Exosomes<br>Cartilage<br>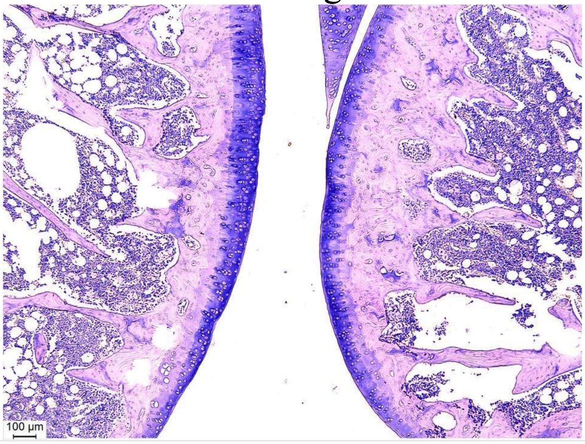 	OA+Exosomes<br>Cartilage<br>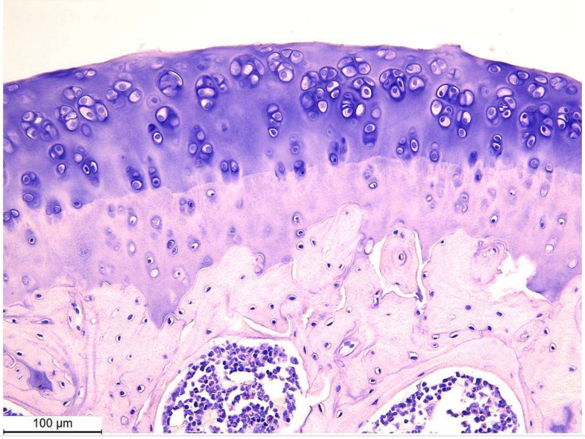
OA+Exosomes<br>Meniscus	OA+Exosomes<br>Infrapatellar fat pad

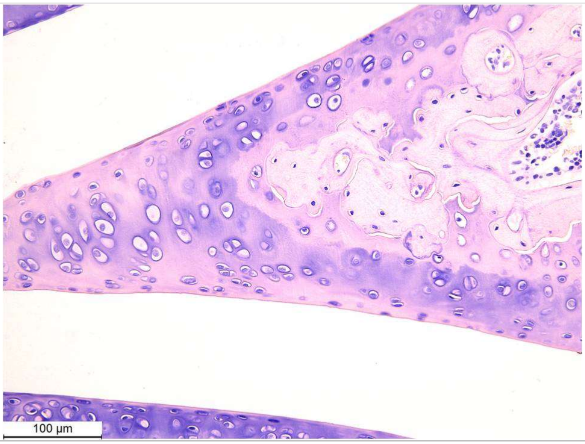	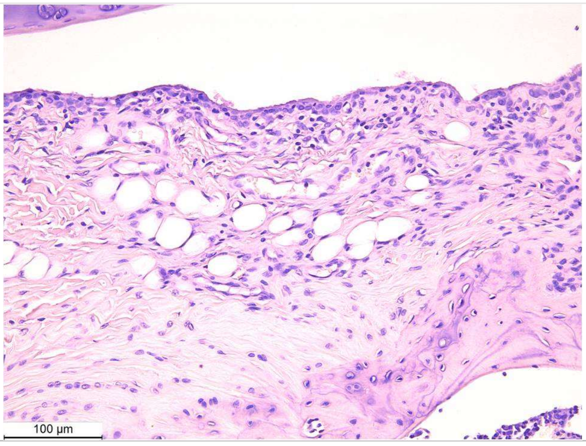
OA+MBV<br>Cartilage<br>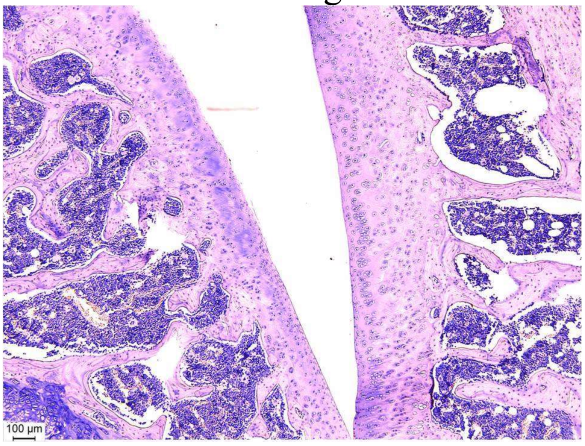	OA+MBV<br>Cartilage<br>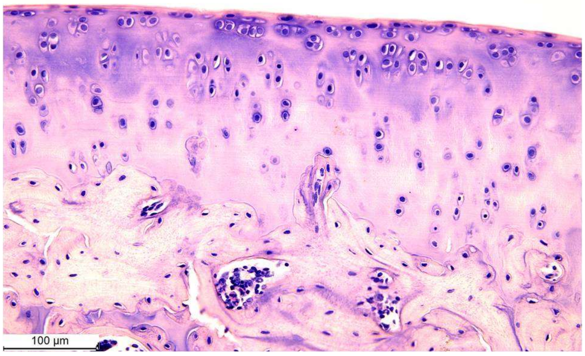
OA+MBV<br>Meniscus<br>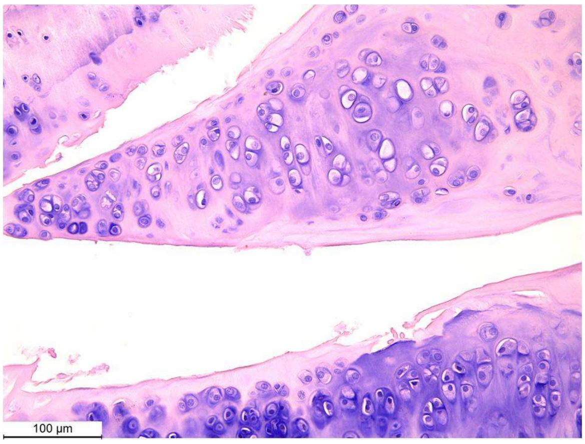	OA+MBV<br>Infrapatellar fat pad<br>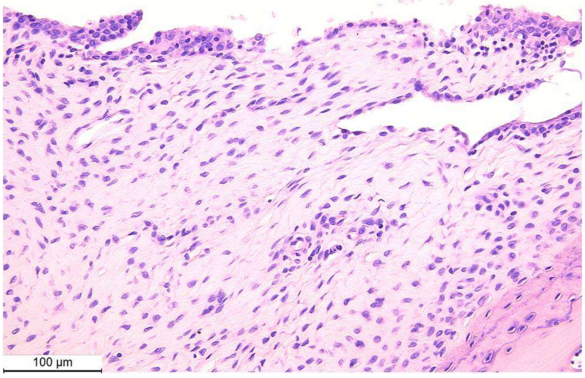
Safranin O/Fast Green staining	
Control	OA

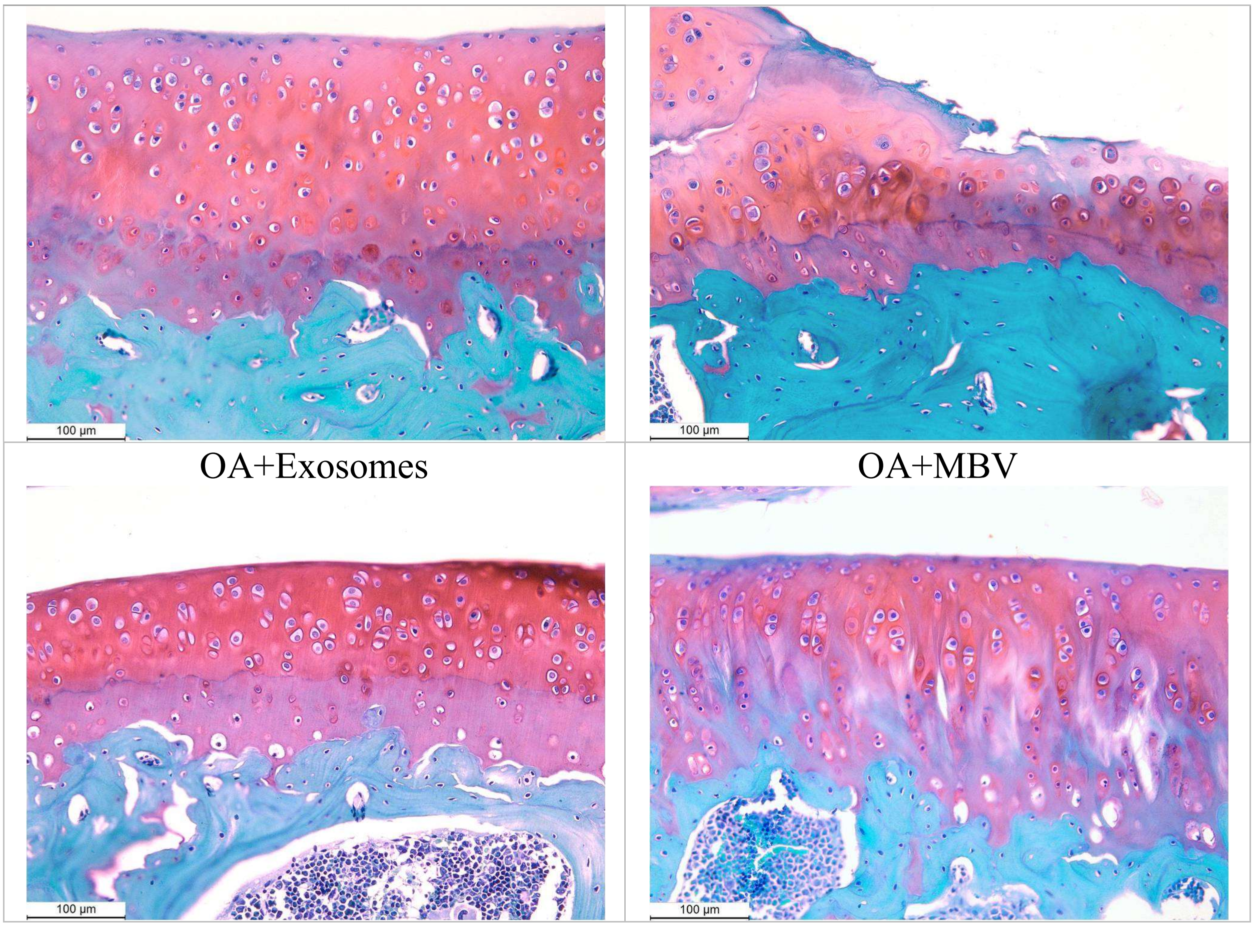

Figure S7. Histochemical analysis of the impact of EVs (exosomes and MBV) from UC-MSC on the progression of pathological changes in cartilage in rats with surgically induced OA. A. Histological staining with H&E, Safranin O/Fast Green. The images depict histological sections of animals 3 weeks post OA therapy. Ctrl - intact control without surgery, OA - OA group injected intra-articularly with PBS, OA+Exo - group with intra-articular injection of EVs from UC-MSC secretome, OA+MBV - group with intra-articular injection of MBV from UC-MSC. Scale bar: 100 µm, magnification 200x

Figure S8. Immunohistochemical analysis of the impact of UC-MSC-derived EVs (exosomes and MBV) on the development of inflammation in cartilage of rats with surgically induced OA.

Figure S8.1., S8.2., S8.3. IHC analysis of inflammatory markers TNF-α, iNOS, Arg-1– markers of enhanced anti-inflammatory microenvironment. Histological tissue images were obtained 3 weeks post-OA therapy. **Figure 8.4.** In situ PCR analysis of NLRP3 inflammasome in joint tissue samples of experimental groups. Ctrl - intact control without surgery, OA - group with surgically induced OA injected with PBS, OA+Exo - group with intra-articular injection of UC-MSC exosomes, OA+MBV - group with intra-articular injection of UC-MSC MBV. Scale bar: 100 µm, magnification 200x

Figure S8.1. Immunohistochemistry TNF-α

Immunohistochemistry TNF-α	
Cartilage	Synovial membrane
Control 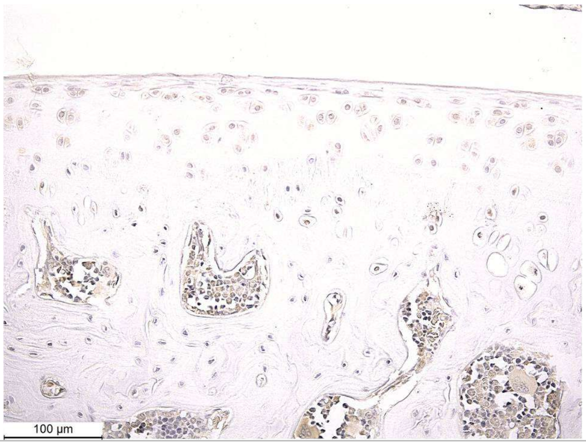	Control 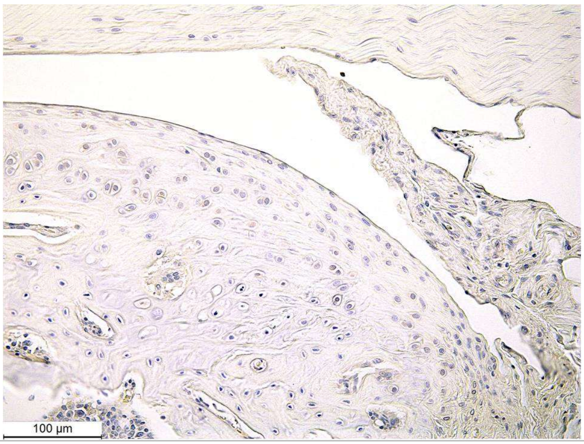
OA 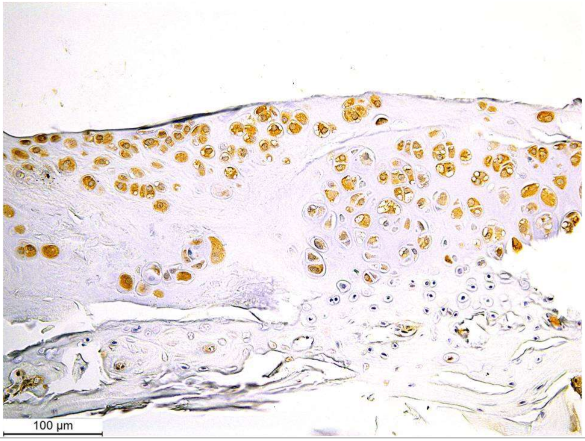	OA 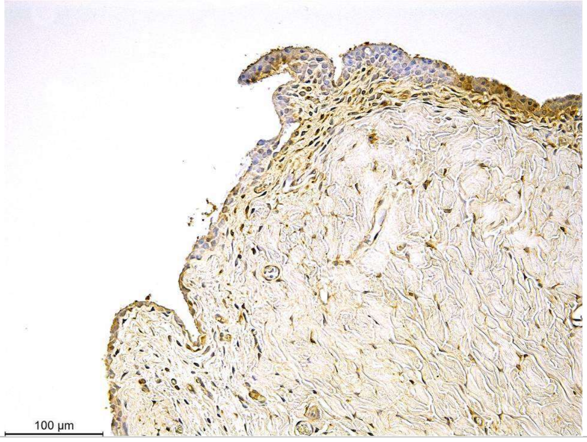
OA+Exosomes 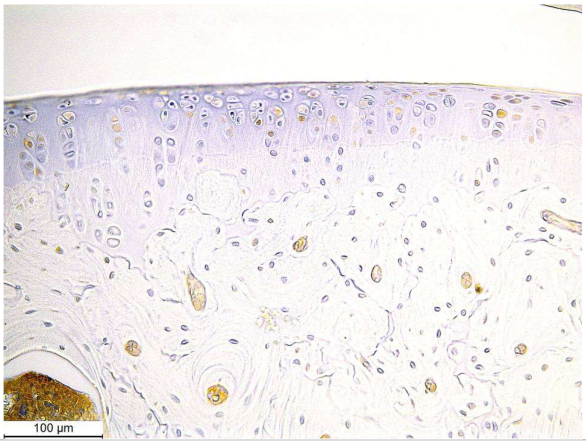	OA+Exosomes 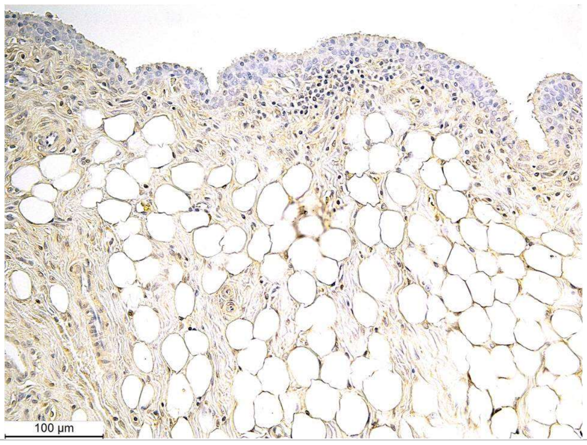

OA+MBV	OA+MBV
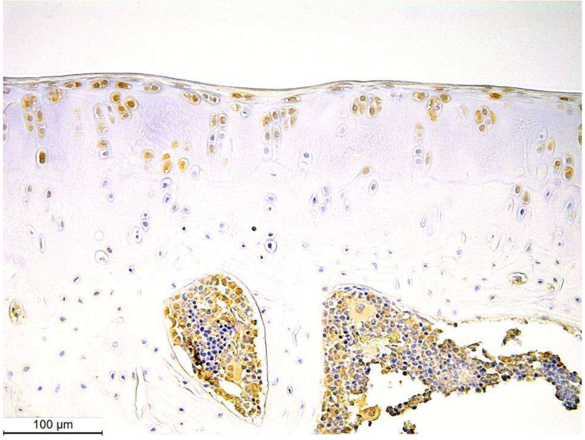	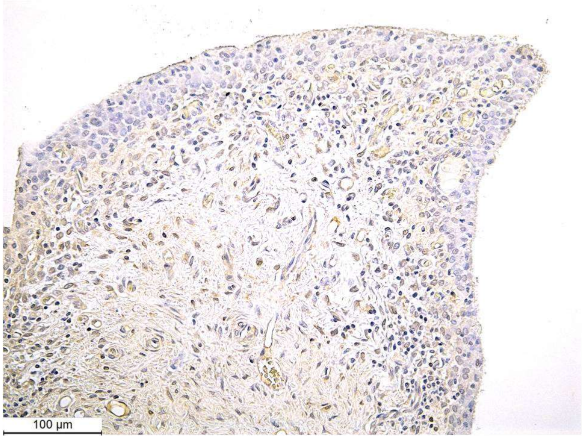

Figure S8.2. Immunohistochemistry iNOS

Immunohistochemistry iNOS	
Cartilage	Synovial membrane
Control	Control
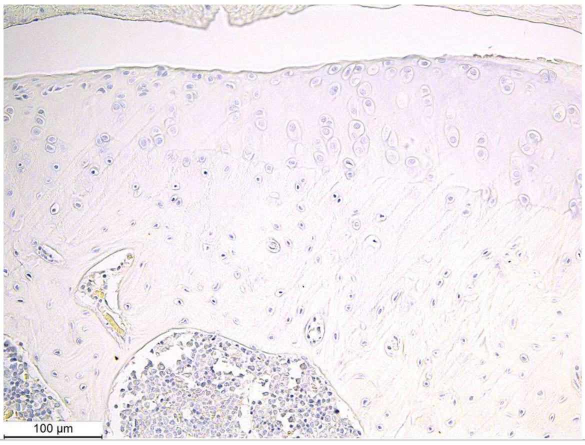	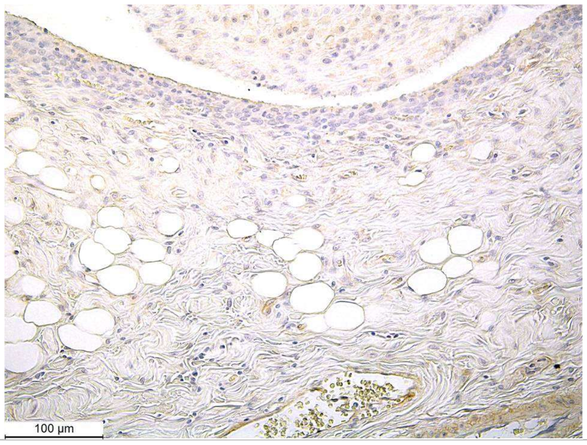
OA	OA
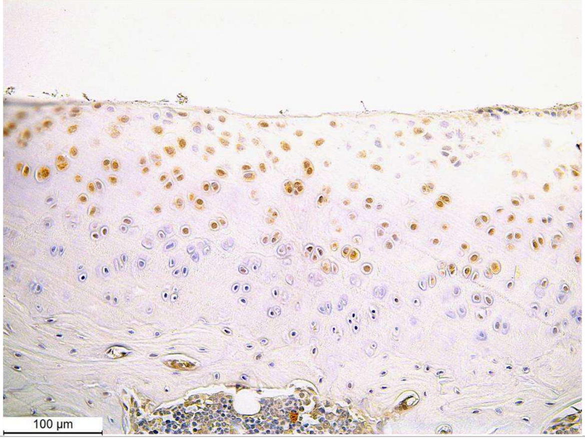	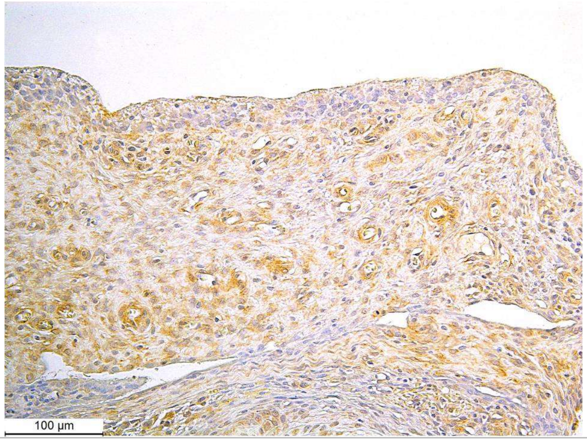

OA+Exosomes	OA+Exosomes
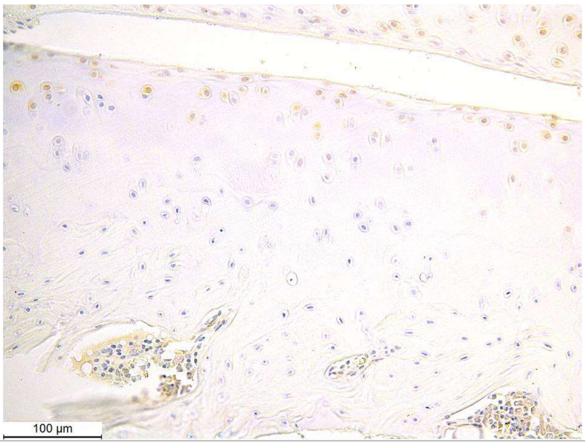	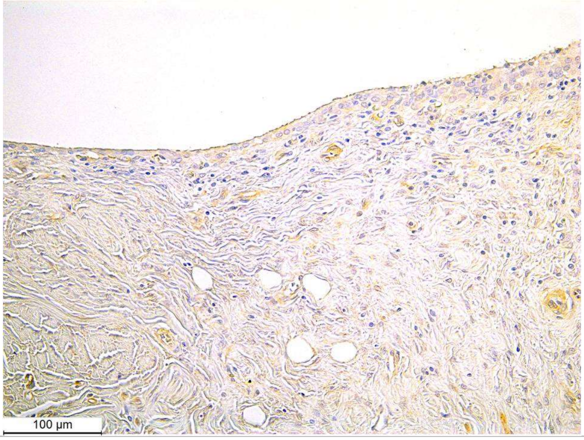
OA+MBV	OA+MBV
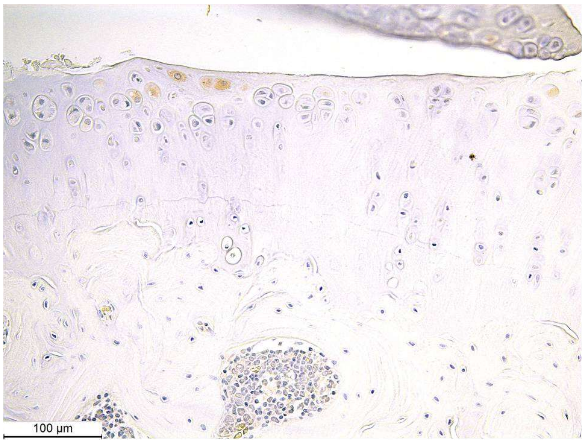	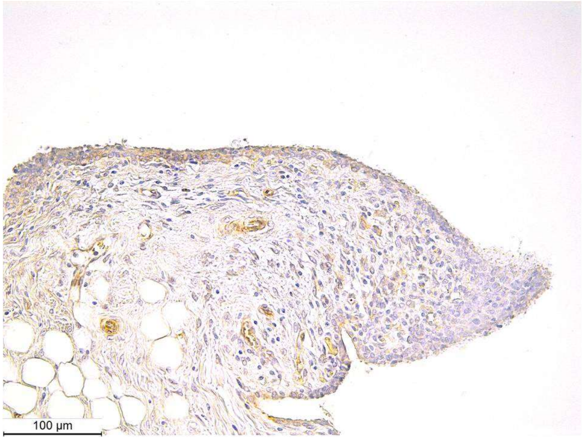

Figure S8.3. Immunohistochemistry Arg1

Immunohistochemistry Arg1	
Cartilage	Synovial membrane
Control	Control
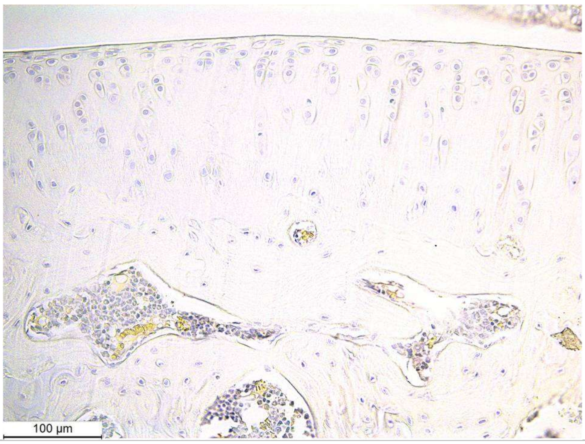	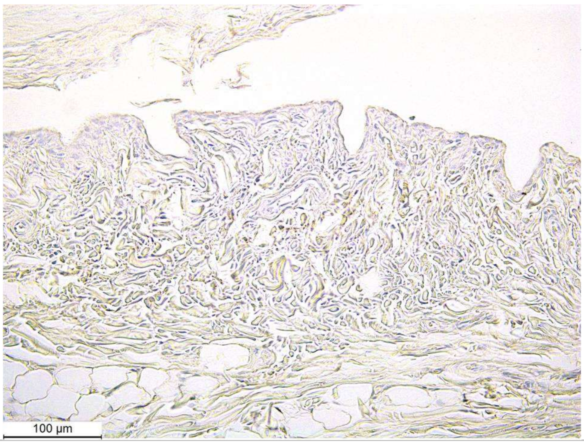

OA	OA
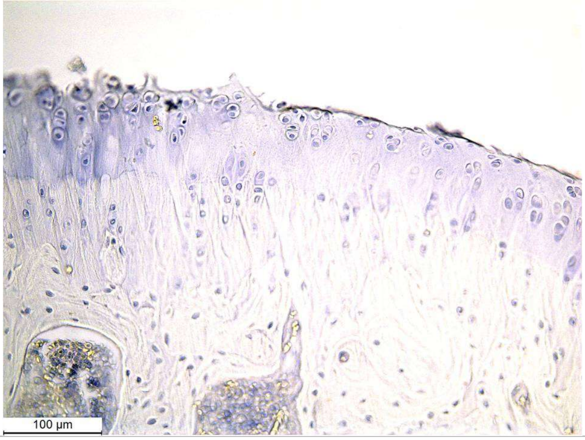	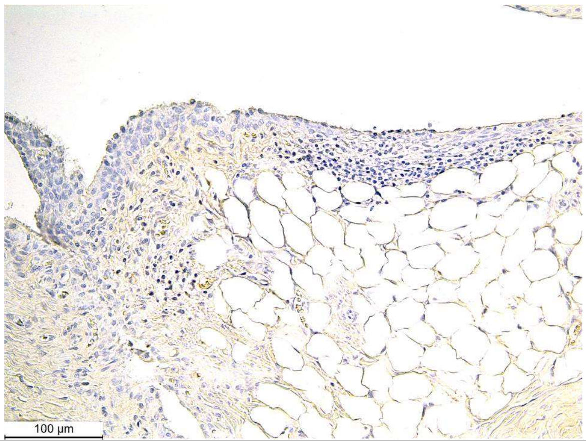
OA+Exosomes	OA+Exosomes
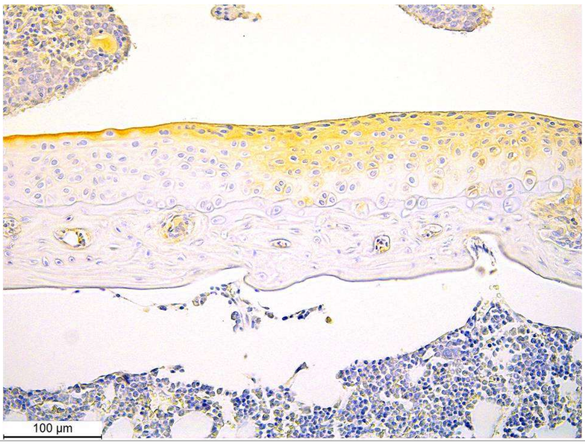	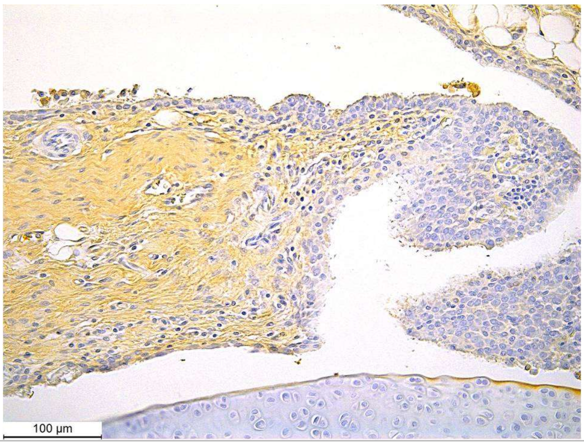
OA+MBV	OA+MBV
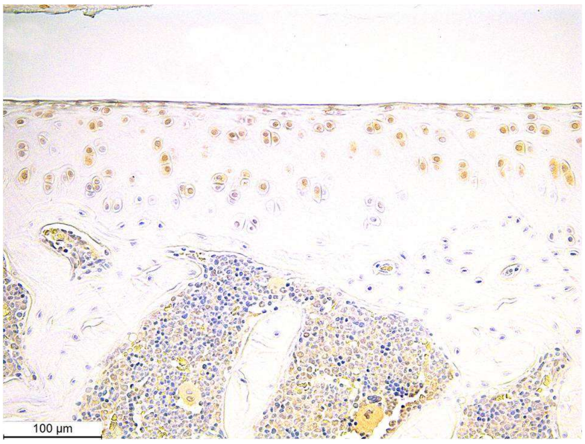	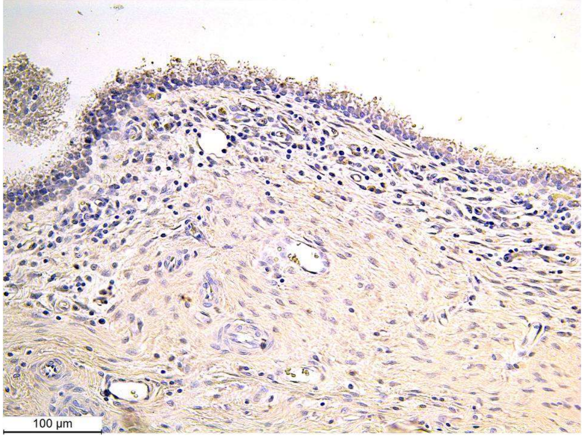

Figure S8.4. PCR in situ NLRP3

PCR in situ NLRP3	
Cartilage	Synovial membrane
Control	Control

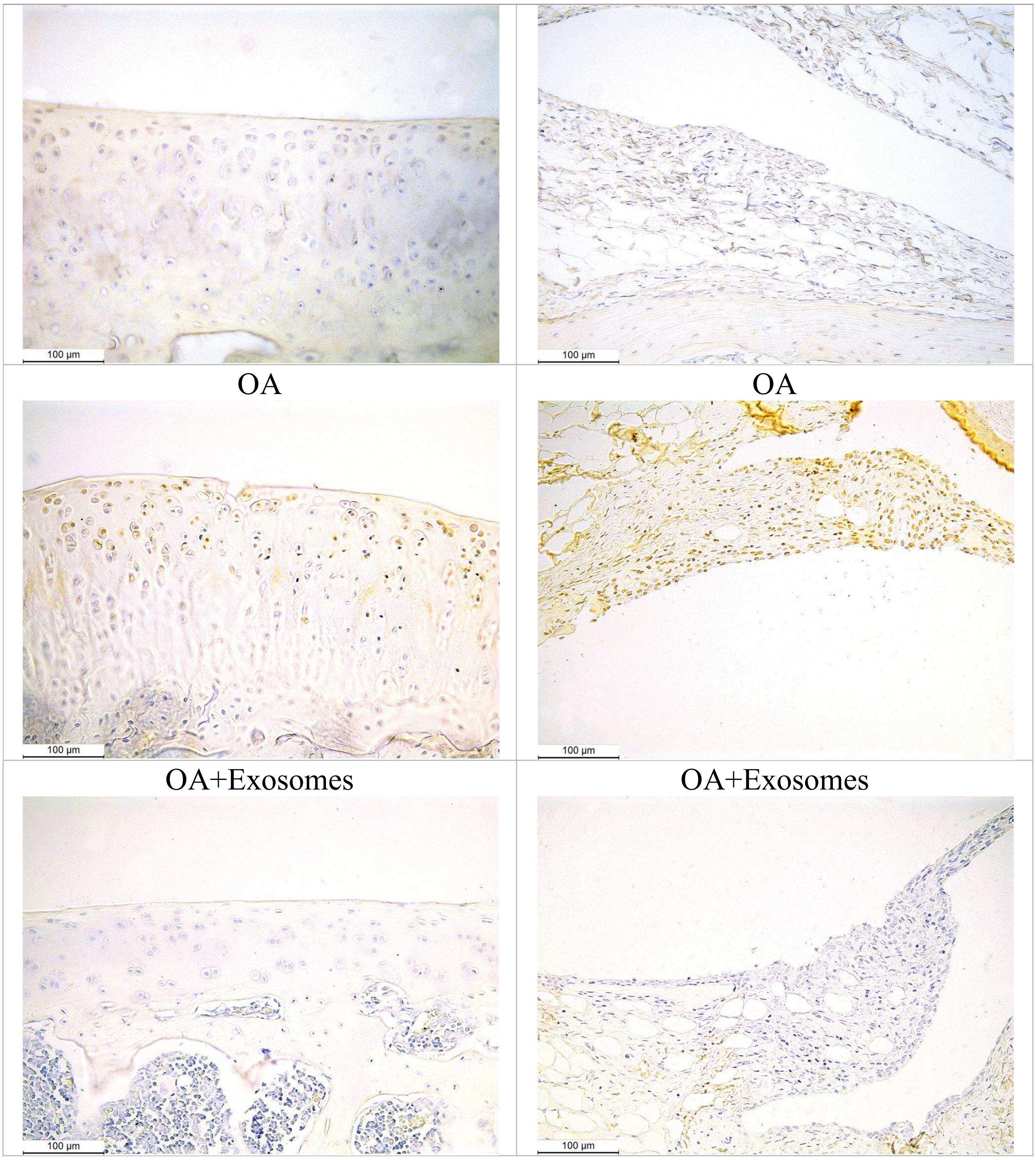
100 µm
100 µm
OA
OA
100 µm
100 µm
OA+Exosomes
OA+Exosomes
100 µm
100 µm

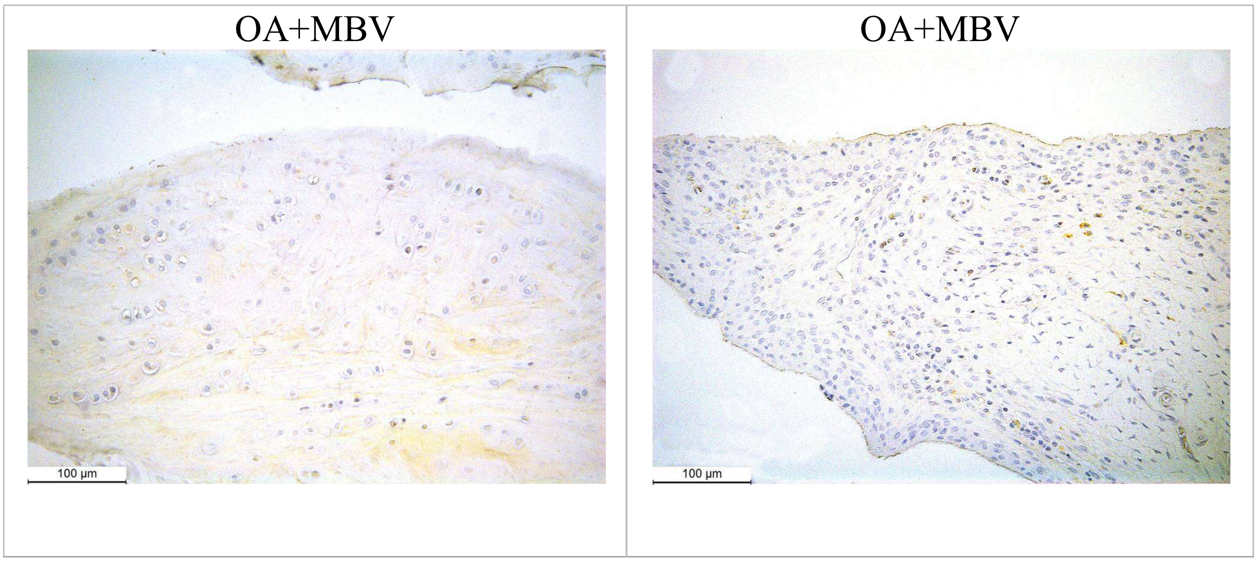

Figure S9. ICH assay of cartilage and infrapatellar fat pad

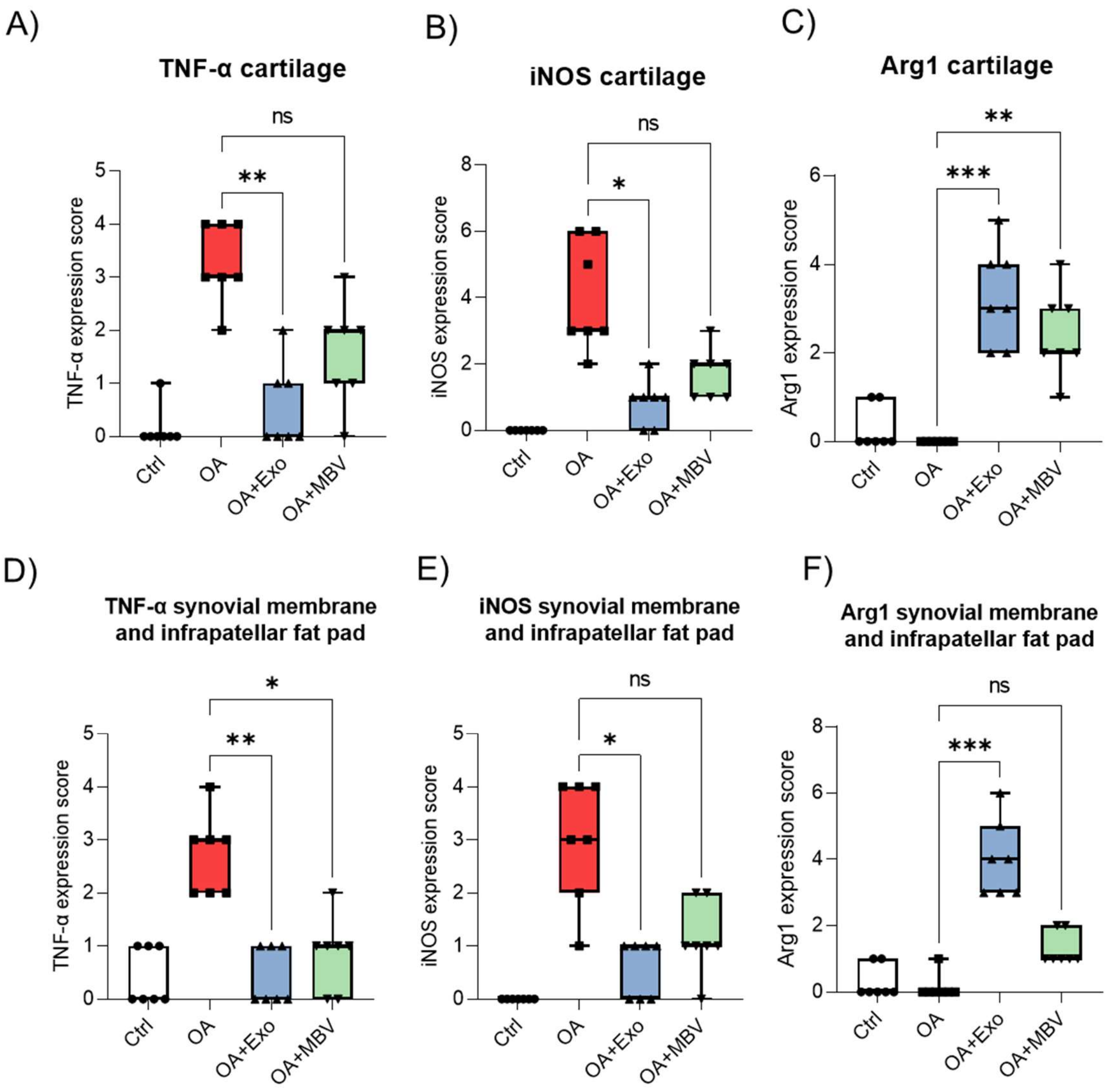

Figure S9. Semi-quantitative scoring of IHC marker expression in joint tissues (cartilage, synovial membrane, infrapatellar fat pad) across different groups.

A. TNF-α cartilage, B. iNOS cartilage, C. Arg1 cartilage, D. TNF-α synovial membrane and infrapatellar fat pad, E. iNOS synovial membrane and infrapatellar fat pad, F. Arg1 synovial membrane and infrapatellar fat pad, $^{*}P < 0.05$; $^{**}P < 0.01$; $^{***}P < 0.001$. The figures (circle, square, triangle) on the graphs indicate the level of expression of IHC marker expression in joint tissues across different groups: ● (control group); ■ (OA group); ▲ (experimental group + exosomes); ▼ (experimental group + MBV).
